# Supplementary material for: Dimensionality reduction of longitudinal ’omics data using modern tensor factorizations
Source: PLoS Comput Biol. 2022 Jul 15;18(7):e1010212. doi: 10.1371/journal.pcbi.1010212 (PMC9328521; doi:10.1371/journal.pcbi.1010212)
Supplement: S1 Text — (PDF) [file pcbi.1010212.s001.pdf]

Dimensionality Reduction of Longitudinal 'Omics Data using Modern Tensor Factorizations. Supplementary discussion

Uria Mor[1,2,*] Yotam Cohen[1,*] Rafael Valdés-Mas[1,*] Denise Kviatcovsky[1]

Eran Elinav[1,3,♯] Haim Avron[2,♯]

[1] Systems Immunology Department, Weizmann Institute of Science, Rehovot, 7610001, Israel.
[2] School of Mathematical Sciences, Tel Aviv University, Tel Aviv 6997801, Israel.
[3] Microbiome and Cancer Division, DKFZ, Heidelberg, Germany.
[*] Equal first contributors.
[♯] Equal last contributors.

All correspondence to:
Haim Avron, Ph.D.
School of Mathematical Sciences, Tel Aviv University
Ramat Aviv,
Tel Aviv, Israel, 6997801
(03) 640-8893 (phone)
haimav@tauex.tau.ac.il
&
Eran Elinav, M.D., Ph.D.
Systems Immunology Department,
Weizmann Institute of Science,
234 Herzl Street,
Rehovot, Israel, 7610001
(08) 934-4014 (phone)
eran.elinav@weizmann.ac.il

Supporting Information

A real order-N tensor $\boldsymbol{\mathcal{A}} \in \mathbb{R}^{d_1 \times d_2 \times \cdots \times d_N}$ is an multi-dimensional array of entries[1]. Each of the entries of $\boldsymbol{\mathcal{A}}$ can be referred to by specifying an N-tuple of numbers $(i_1, ..., i_N)$ where $i_k \in \{1, 2, \ldots, d_k\}$.

[1]In pure mathematics this is actually the definition of a *hypermatrix*, while a *tensor* is an algebraic object that describes a multilinear relationship. However, in data science often the term 'tensor' is abused to mean a hypermatrix, and we adopt this terminology here as well.

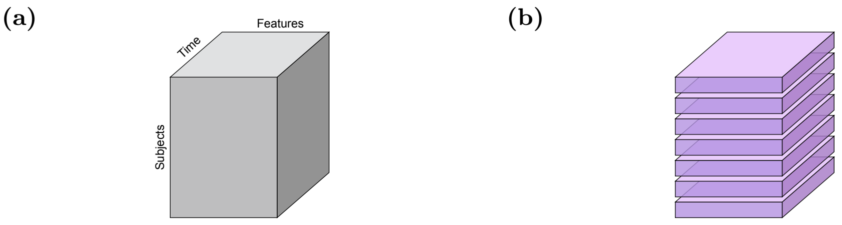

Figure 1: Subject centered view of 3rd order tensor . Fig. 1a An illustration of the data structure. Fig. 1b The right panel presents a breakdown of the left tensor into m horizontal slices that are $p \times n$ matrices.

A third-order tensor $\mathcal{A} \in \mathbb{R}^{m \times p \times n}$ can also be viewed as an m elements long list of $p \times n$ matrices, each an horizontal slice of the tensor (Fig. 1a and 1b). This mathematical construct is appealing in the context of longitudinal studies as it enables storing the data in a way that is consistent with the data collection. One might think of $\mathcal{A}$ as a data-structure for holding the results of an experiment during which n samples were collected from m participants, and each sample is characterized by p features. These features may be genes in the case of RNA-seq samples, taxonomic composition of shotgun metagenomics sequencing, etc. The tensor data structure reflects not only the data points but also key relationships between them. For example, let $1 \leq i \leq p$ an integer denoting the index of a certain feature, then variations of this feature across the whole cohort are obtained by fixing the second coordinate of the tensor to i: $\mathcal{A}_{:,i,:}$[2]. Similarly, tracking this feature in a single timepoint t is done by restriction of the last two coordinates $\mathcal{A}_{:,i,t}$.

The arrangement of the same data in the form of a matrix, which has only two dimensions, would require us to make a somewhat arbitrary choice about which of the two dimensions are to be coalesced into a single dimension. For example, one might consider each individual subject as a single sample, and as such each individual will have a designated row in the data matrix, while repeated measurements of the same feature at several timepoints are treated as entirely different features, resulting in an $m \times np$ matrix. Note that by concatenating the repeated samples of each individual, we form a new feature space in which the temporal context is lost. Another option is to define samples as p dimensional entities measured for each subject at all timepoints, resulting in an $nm \times p$ matrix. This formulation breaks the correspondence between data of the same individual across timepoints, as well as the data of all individuals at a single timepoint.

Continuing with the above example, if in addition we were to sample each subject at k body sites (instead of one), then the number of possible ways of rearranging data in the form of a matrix would have increased to four different choices, each of which captures a different aspect of the experimental design. On the other hand, having the data in a format of tensor would only require an addition of a single mode for describing the k different body sites, resulting in a fourth-order tensor in $\mathbb{R}^{m \times p \times n \times k}$.

The advantage of representing data in native tensor format as opposed to *matricizing* it is not unique to longitudinal studies and appears in many domains. See [1] for discussion.

In this work, we are concerned with tensor-based dimensionality reduction methods. It was previously shown, e.g. [2] and more recently [3, 4], that tensor-based dimensionality reduction methods have a potential to provide more meaningful output compared to analogous matrix-based methods. Most of these works consider the low-rank approximation in the form of a CP factorization [5]

$$\mathcal{A} \approx \mathcal{A}_r = \sum_{i=1}^{r} \sigma_i \mathbf{u}_i^{(1)} \circ \mathbf{u}_i^{(2)} \circ \ldots \circ \mathbf{u}_i^{(N)} \in \mathbb{R}^{d_1 \times \ldots \times d_N}, \tag{1}$$

where σ_i are positive scalers, the *components* $\mathbf{u}_i^{(j)}$ are d_j dimensional unit vectors and $\circ$ denotes the outer-product operation ($\mathbf{u}_i^{(j)} \circ \mathbf{u}_i^{(k)}$ is a $d_j \times d_k$ matrix, while $\mathbf{u}_i^{(j)} \circ \mathbf{u}_i^{(k)} \circ \mathbf{u}_i^{(l)}$ is a tensor in $\mathbb{R}^{d_j \times d_k \times d_l}$). The number of terms r in the above factorization denotes the maximal rank of the sought approximate $\mathcal{A}_r$ of the original tensor $\mathcal{A}$. For brevity, we denote a CP factorization by $\mathcal{A}_r = [\mathbf{\Sigma}; \mathbf{U_1}, ..., \mathbf{U_N}]$ where $\mathbf{\Sigma} = \text{diag}(\sigma_1, ..., \sigma_\mathbf{r})$, and $\mathbf{U}_j$ are $d_j \times r$ matrices of columns that are the unit vectors $\mathbf{u}_1^{(j)}, ...\mathbf{u}_r^{(j)}$. As a convention, each of the r summands $(\sigma_i \mathbf{u}_i^{(1)} \circ ... \circ \mathbf{u}_i^{(N)})$ is a rank-1 tensor (for alternative definitions of tensor rank see [6]). This factorization provides an intuitive breakdown of the data which is somewhat analogous to that of a PCA for matrix data: the overall contribution of each component $\mathbf{u}_i^{(j)}$ to the approximation is determined by the magnitude of the corresponding scaler σ_i (larger scaler implies greater contribution), and the components themselves may reflect the different modalities of the data (in the above

[2]Here, we are using Matlab notation, in which ':' denotes the entire range of a mode, and '$l : k$' denotes $\{l, l+1, \ldots, k\}$.

example, $\mathbf{u}_i^{(1)}$ are associated with the different subjects, while $\mathbf{u}_i^{(2)}, \mathbf{u}_i^{(3)}$ components are associated with features and timepoints respectively).

Computing an approximation in the form of a CP decomposition of a given tensor $\mathcal{A}$ is usually accomplished by solving the following optimization problem:

$$[\mathbf{\Sigma}; \mathbf{U_1}, ..., \mathbf{U_N}] \quad = \arg\min_{\tilde{\mathbf{\Sigma}}, \tilde{\mathbf{U}}_1, ..., \tilde{\mathbf{U}}_N} \|\mathcal{A} - \textstyle\sum_{i=1}^{r} \tilde{\sigma}_i \tilde{\mathbf{u}}_i^{(1)} \circ ... \circ \tilde{\mathbf{u}}_i^{(N)}\|_F^2 \tag{2}$$

where $\|\mathcal{X}\|_F$ denotes the Frobenius norm of the tensor $\mathcal{X}$:

$$\|\mathcal{X}\|_F^2 = \sum_{i_1=1}^{d_1} \cdots \sum_{i_N=1}^{d_N} \mathcal{X}_{i_1, i_2, \dots i_N}^2$$

The current gold-standard method for solving this problem is Alternating Least Squares (ALS) [6].

It is generally hard to solve Problem (2) as it is non-convex, thus potentially having many local minimizers which are not global minima. Indeed, even the gold-standard method (ALS) is not guaranteed to find a global minimum. Moreover, given a data tensor $\mathcal{A}$ (subjects, features and time), and its CP form approximate $\mathcal{A}_r = [\mathbf{\Sigma}; \mathbf{U_1}, \mathbf{U_2}, \mathbf{U_3}]$, suppose that we are provided with data for a new participant $\mathcal{X} \in \mathbb{R}^{1 \times p \times n}$, then the task of extending the current factorization to the new data tensor, which is $\mathcal{A}$ augmented with the new sample $\mathcal{X}$, is far from a trivial one. Such out-of-sample extension capability is a fundamental requirement from any embedding algorithm that we wish to use as a step in a ML pipeline.

The aim of TCAM is to provide a tensor-based PCA-like tool that is better than CP, in terms of ease of interpretation of the model's outcomes and mathematical properties that ensure safe application to downstream statistical analysis and ML workflows.

A PCA

Since we strive to have to claim that TCAM is 'PCA-like', it is useful to first present a brief definition of PCA, and discuss it characteristics. All constructions, definitions and properties presented in this section are taken from [7].

Let $\mathbf{A} \in \mathbb{R}^{m \times p}$ be a data matrix with rows $\mathbf{a}_1, \dots, \mathbf{a}_m$ corresponding to m samples, and assume that $\mathbf{A}$ has been centered (so the column means are zero). PCA is defined by an orthogonal linear transformation $\mathbf{W}$ transforming the rows of $\mathbf{A}$ to a new coordinates system, in which, the largest portion of variation in the data lies on the first axis (called the first principal component of $\mathbf{A}$), i.e. $\mathbf{z}_1 = \mathbf{A}\mathbf{w}_1$ where $\mathbf{w}_1 \in \mathbb{R}^p$ is the maximizer of $\mathbf{w^T A^T A w}$ subjected to $\|\mathbf{w}\|_2^2 = 1$. The kth largest portion of variation lies on the kth principal component of $\mathbf{A}$, i.e.

$$\mathbf{z}_k = \mathbf{A}\mathbf{w}_k, \qquad\qquad \mathbf{w}_k = \text{argmax}_{\|\mathbf{w}\|_2=1} \mathbf{w^T} {\mathbf{A}^{(k)}}^{\mathbf{T}} \mathbf{A}^{(k)} \mathbf{w}$$

where given the first $k-1$ principal components of $\mathbf{A}$ we define $\mathbf{A}^{(k)} = \mathbf{A}(\mathbf{I} - \sum_{i=1}^{k-1} \mathbf{w}_i \mathbf{w}_i^{\mathbf{T}})$. The complete factorization yields the expression $\mathbf{Z} = \mathbf{AW}$, where rows of the matrix $\mathbf{Z} \in \mathbb{R}^{m \times p}$ are called the *sample PC scores* (the ith row of $\mathbf{Z}$ contains the sample PC scores of the ith sample $\mathbf{a}_i$). The orthogonal $p \times p$ matrix $\mathbf{W}$ is called the weight or coefficients matrix. The rank k-truncated PCA of a $m \times p$ matrix $\mathbf{A}$ is defined by

$$\mathbf{Z}_k = \mathbf{A}\mathbf{W}_k \in \mathbb{R}^{m \times k} \tag{3}$$

where $\mathbf{W}_k := [\mathbf{w}_1, ..., \mathbf{w}_k]$ is an $p \times k$ matrix with orthonormal rows; $\mathbf{W}_k^{\mathbf{T}} \mathbf{W}_k = \mathbf{I}_k$. The k-truncated PCA $\mathbf{Z}_k$ consists of only the first k principal components.

The **sample variance-covariance matrix** of the collection $\{\mathbf{a}_i\}_{i=1}^m$ of m, (centered) p-dimensional samples $\mathbf{S}$ is given by $\mathbf{S} = (m-1)^{-1} \mathbf{A^T A}$.

Property A.1 ([7, Chapter 2, Properties A1 and A2]). *For any integer $q = 1, \dots, p$, consider the transformation $\mathbf{y}_i = \mathbf{B^T a}_i$, where $\mathbf{y} \in \mathbb{R}^q$ and $\mathbf{B}$ is a $p \times q$ matrix with q orthonormal columns. Define $\mathbf{S_y} = \mathbf{B^T S B} \in \mathbb{R}^{q \times q}$ the variance-covariance matrix for $\mathbf{y}$. The variance component of $\mathbf{S_y}$, quantified by* $\text{Tr}(\mathbf{S_y})$*, is maximized when taking $\mathbf{B} = \mathbf{W}_q$ and minimized when $\mathbf{B} = [\mathbf{w}_{p-q}, \mathbf{w}_{p-q+1}, ...\mathbf{w}_p]$*

Property A.2 ([7, Chapter 3, Eq. 3.1.4, Property A3]). $\mathbf{S} = \sum_{i=1}^p \lambda_i \mathbf{w}_i \mathbf{w}_i^{\mathbf{T}}$

Property A.3 ([7, Chapter 3, Property G4]). *Let $\tilde{\mathbf{A}} = [\mathbf{a}_1; ...; \mathbf{a}_m]$ be a $m \times p$ matrix of m, p-dimensional observations. Define $\mathbf{A}$ to be the $m \times p$ matrix whose i^{th} row is $\mathbf{a}_i - \bar{\mathbf{a}}$ where $\bar{\mathbf{a}} = \frac{1}{m} \sum_{j=1}^m \mathbf{a}_j$ and consider the matrix $\mathbf{A A^T} \in \mathbb{R}^{m \times m}$. The ith diagonal element of $\mathbf{AA^T}$ is the squared Euclidean distance of the sample $\mathbf{a}_i$ from the point $\bar{\mathbf{a}}$ that is the center of gravity of the points $\mathbf{a}_1 ... \mathbf{a}_m$. Also, the i, j entry of $\mathbf{AA^T}$, given by $\langle \mathbf{a}_i - \bar{\mathbf{a}}, \mathbf{a}_j - \bar{\mathbf{a}} \rangle$, is the cosine of the angle between the lines joining the points $\mathbf{a}_j$ and $\mathbf{a}_i$ to $\bar{\mathbf{a}}$, scaled by the distances of $\mathbf{a}_j$ and $\mathbf{a}_i$ from $\bar{\mathbf{a}}$. Suppose that $\mathbf{a}_1 ... \mathbf{a}_m$ are projected to a q-dimensional subspace using a linear orthogonal transformation $\mathbf{y}_i = \mathbf{B^T a}_i$, and let $\mathbf{Y} := [\mathbf{y}_1 - \bar{\mathbf{y}}; ...; \mathbf{y}_m - \bar{\mathbf{y}}] \in \mathbb{R}^{m \times q}$ for $\bar{\mathbf{y}} = m^{-1} \sum_{j=1}^m \mathbf{y}_j \in \mathbb{R}^q$. Then the choice $\mathbf{B} = \mathbf{W}_q$ minimizes the distortion $\|\mathbf{YY^T} - \mathbf{AA^T}\|_F^2$*

Singular Value Decomposition. One practical way of describing and computing PCA for a matrix $\tilde{\mathbf{A}} \in \mathbb{R}^{m\times p}$, is using the singular value decomposition of its column centered form. Let again $\mathbf{A}$ denote the column centered form of $\tilde{\mathbf{A}}$, and let $\mathbf{A} = \mathbf{U}\Sigma\mathbf{V}^{\mathbf{T}}$ be its Singular Value Decomposition (SVD). That is, $\mathbf{U} \in \mathbb{R}^{m\times m}$ and $\mathbf{V} \in \mathbb{R}^{p\times p}$ are orthonormal matrices, and $\Sigma \in \mathbb{R}^{m\times p}$ is a matrix with non-negative elements $\sigma_1 \geq \sigma_2 \geq ...$ on its main diagonal and zeros elsewhere. The sample variance-covariance matrix $\mathbf{S}$ can rewritten as $\mathbf{S} = (m-1)^{-1}\mathbf{V}\Sigma^{\mathbf{T}}\Sigma\mathbf{V}^{\mathbf{T}}$, thus the columns of $\mathbf{V}$ are the eigenvectors of $\mathbf{S}$ and, according to Property A.2, the coefficients in the PCA decomposition of $\tilde{\mathbf{A}}$.

Let us denote the rank-r truncated SVD of $\mathbf{A}$ by $\mathbf{A}_r = \mathbf{U}_r\Sigma_r\mathbf{V}_r^{\mathbf{T}}$ where $\mathbf{U}$ and $\mathbf{V}$ are the matrices obtained be taking the first r columns of $\mathbf{U}$ and $\mathbf{V}$ respectively, and $\Sigma_r = \mathrm{diag}(\sigma_1, ..., \sigma_r)$. Suppose that the rank of $\mathbf{A}$ is greater or equal to r, then the rank of $\mathbf{A}_r$ is exactly r. This truncation of the SVD enjoys many algebraic properties, one such major result is the Eckart-Young-Mirsky Theorem stating that the r-rank truncated SVD of a matrix is in a sense the best approximate of rank lower or equal to r:

$$\mathbf{A}_r = \arg\min_{\tilde{\mathbf{A}}, \mathrm{rank}(\tilde{\mathbf{A}})\leq r} \|\mathbf{A} - \tilde{\mathbf{A}}\|_F^2$$

This result directly implies Properties A.1 and A.3 of the PCA that are concerned with the maximization of the variance and minimizing the distortion of the projected configuration. Thus, we see that the SVD can be used not only as an alternative construction algorithm for the PCA, but also, due to Eckart-Young Theorem, can serve as the mathematical justification to some of the PCA's key properties.

B Tensor Component Analysis

A recent work by Kilmer et. al. introduced a tensor version of the Eckart-Young Theorem [1], stating that the truncated tensor SVD is the best low rank approximate within a specified tensor-tensor product framework based on the $\star_{\mathrm{M}}$-product. The $\star_{\mathrm{M}}$-product operation is defined by an invertible matrix $\mathbf{M}$, and the best low rank approximation results from [1] were established for matrices $\mathbf{M}$ that are nonzero multiple of a unitary matrix ($\mathbf{M}^{\mathbf{T}}\mathbf{M} = \mathbf{M}\mathbf{M}^{\mathbf{T}} = c^2\mathbf{I}_n$ for some constant $c \neq 0$). In this work, we only consider $\mathbf{M}$ that are unitary matrices.

We construct the TCAM on top the tensor SVD introduced by Kilmer et. al., and utilize Kilmer's Eckart-Young-like result to establish tensor analogs of Properties A.1 and A.3, in the same way that matrix SVD can be used for deriving the same properties for matrices. As a prelude to our discussion, we present some key notions and operations necessary for our derivation. Elaborated introduction and discussion can be found in [1].

B.1 Tensor-tensor $\star_{\mathrm{M}}$-product framework

We begin with some definitions. Let $\mathbf{M}$ be an $n \times n$ orthogonal matrix ($\mathbf{M}\mathbf{M}^{\mathbf{T}} = \mathbf{I}_n = \mathbf{M}^{\mathbf{T}}\mathbf{M}$), and a tensor $\mathcal{A} \in \mathbb{R}^{m\times p\times n}$. We define the **domain transform** specified by $\mathbf{M}$ as $\widehat{\mathcal{A}} := \mathcal{A} \times_3 \mathbf{M}$, where $\times_3\mathbf{M}$ denotes the tensor-matrix multiplication of applying $\mathbf{M}$ to each of the tensor n dimensional tubal fibers ($\mathcal{A}_{i,j,:}$). The **transpose** of a real $m \times p \times n$ tensor $\mathcal{A}$ with respect to $\mathbf{M}$, denoted by $\mathcal{A}^{\mathbf{T}}$, is a $p \times m \times n$ tensor for which $[\widehat{\mathcal{A}^{\mathbf{T}}}]_{:,:,i} = [\widehat{\mathcal{A}}^{\mathbf{T}}]_{:,:,i} = [\widehat{\mathcal{A}}]_{:,:,i}{}^{\mathbf{T}}$. Given two tensors $\mathcal{A} \in \mathbb{R}^{m\times p\times n}$ and $\mathcal{B} \in \mathbb{R}^{p\times l\times n}$, the facewise tensor-tensor product of $\mathcal{A}$ and $\mathcal{B}$, denoted by $\mathcal{A} \vartriangle \mathcal{B}$, is the $m \times l \times n$ tensor for which $[\mathcal{A} \vartriangle \mathcal{B}]_{:,:,i} = \mathcal{A}_{:,:,i}\mathcal{B}_{:,:,i}$.

The tensor-tensor $\star_{\mathrm{M}}$**-product** of $\mathcal{A} \in \mathbb{R}^{m\times p\times n}$ and $\mathcal{B} \in \mathbb{R}^{p\times l\times n}$ is defined by $\mathcal{A} \star_{\mathrm{M}} \mathcal{B} := (\widehat{\mathcal{A}} \vartriangle \widehat{\mathcal{B}}) \times_3 \mathbf{M}^{-1} \in \mathbb{R}^{m\times l\times n}$. A few definitions now naturally follow. The $p \times p \times n$ **identity tensor** with respect to $\star_{\mathrm{M}}$, is the tensor $\mathcal{J}$ such that for any tensor $\mathcal{E} \in \mathbb{R}^{p\times p\times n}$ it holds that $\mathcal{J} \star_{\mathrm{M}} \mathcal{E} = \mathcal{E} = \mathcal{E} \star_{\mathrm{M}} \mathcal{J}$. In situations where the dimensions are unclear from context, we use $\mathcal{J}_m$ to denote the $m \times m \times n$ identity tensor. Two tensors $\mathcal{A}, \mathcal{B} \in \mathbb{R}^{1\times m\times n}$ are called $\star_{\mathrm{M}}$**-orthogonal slices** if $\mathcal{A}^{\mathbf{T}} \star_{\mathrm{M}} \mathcal{B} = \mathbf{0}$, where $\mathbf{0} \in \mathbb{R}^{1\times 1\times n}$ is the zero tube fiber, while $\mathcal{Q} \in \mathbb{R}^{m\times m\times n}$ is called $\star_{\mathrm{M}}$**-unitary** if $\mathcal{Q}^{\mathbf{T}} \star_{\mathrm{M}} \mathcal{Q} = \mathcal{J} = \mathcal{Q} \star_{\mathrm{M}} \mathcal{Q}^{\mathbf{T}}$.

The following definition is new, and is important for stating the PCA-like properties of TCAM.

Definition B.1. *A tensor $\mathcal{B} \in \mathbb{R}^{p\times k\times n}$ is said to be a* **pseudo $\star_{\mathrm{M}}$-unitary tensor** *(or* **pseudo $\star_{\mathrm{M}}$-orthogonal***) if $\mathcal{B}^{\mathbf{T}} \star_{\mathrm{M}} \mathcal{B}$ is f-diagonal (i.e., all frontal slices are diagonal), and all frontal slices of $(\mathcal{B}^{\mathbf{T}} \star_{\mathrm{M}} \mathcal{B}) \times_3 \mathbf{M}$ are diagonal matrices with entries that are either ones or zeros.*

B.2 The TSVDM

With the $\star_{\mathrm{M}}$-product framework set-up, it is now possible to introduce the tensor singular value decomposition (TSVDM). Let $\mathcal{A} \in \mathbb{R}^{m\times p\times n}$ be a real tensor, then is possible to write the full **tubal singular value decomposition** of $\mathcal{A}$ as $\mathcal{A} = \mathcal{U} \star_{\mathrm{M}} \mathcal{S} \star_{\mathrm{M}} \mathcal{V}^{\mathbf{T}}$, where $\mathcal{U}, \mathcal{V}$ are $m \times m \times n$ and $p \times p \times n$ $\star_{\mathrm{M}}$-unitary tensors respectively, and $\mathcal{S} \in \mathbb{R}^{m\times p\times n}$ is an **f-diagonal** tensor, that is, a tensor whose frontal slices ($\mathcal{S}_{:,:,i}$) are

matrices with zeros outside their main diagonal (see [1] for additional details). We use the notation $\hat{\sigma}_j^{(i)}$ do denote the jth largest singular value on the ith lateral face of $\widehat{\mathcal{S}}$: $\hat{\sigma}_j^{(i)} := \widehat{\mathcal{S}}_{j,j,i}$.

The TSVDM construction makes it possible to expand the concept of tensor-rank discussed earlier. The **t-rank** of $\mathcal{A}$ is the number of nonzero tubes of $\mathcal{S}$: $r = |\left\{ i = 1, \ldots, n \ ; \ \|\mathcal{S}_{i,i,:}\|_F^2 > 0 \right\}|$. Additionally, the **multi-rank** of $\mathcal{A}$ under $\star_{\mathrm{M}}$, denoted by the vector $\boldsymbol{\rho} \in \mathbb{N}^n$ whose i^{th} entry is $\boldsymbol{\rho}_i = \mathrm{rank}(\widehat{\mathcal{A}}_{:,:,i})$, and the **implicit rank** under $\star_{\mathrm{M}}$ of a tensor $\mathcal{A}$ with multi-rank $\boldsymbol{\rho}$ under $\star_{\mathrm{M}}$ is $r = \sum_{i=1}^{n} \boldsymbol{\rho}_i$.

The definitions of tensor t-rank and multi-rank under $\star_{\mathrm{M}}$ also make it possible to define TSVDM rank truncation with respect to these ranks. The tensor $\mathcal{A}^{(q)} = \mathcal{U}_{:,1:q,:} \star_{\mathrm{M}} \mathcal{S}_{1:q,1:q,:} \star_{\mathrm{M}} \mathcal{V}_{:,1:q,:}{}^{\mathbf{T}}$ denotes the **t-rank q truncation** of $\mathcal{A}$ under $\star_{\mathrm{M}}$, while multi-rank $\boldsymbol{\rho}$ truncation of $\mathcal{A}$ under $\star_{\mathrm{M}}$ is given by the tensor $\mathcal{A}_{\boldsymbol{\rho}}$ for which $\widehat{\mathcal{A}_{\boldsymbol{\rho}}}_{:,:,i} = \widehat{\mathcal{U}}_{:,1:\boldsymbol{\rho}_i,i} \widehat{\mathcal{S}}_{1:\boldsymbol{\rho}_i,1:\boldsymbol{\rho}_i,i} \widehat{\mathcal{V}}^{\mathbf{T}}_{:,1:\boldsymbol{\rho}_i,i}$. Note that for t-rank truncation the $\mathcal{U}$ and $\mathcal{V}$ factors are $\star_{\mathrm{M}}$-orthogonal, while for multi-rank truncation they are only pseudo $\star_{\mathrm{M}}$-orthogonal.

The Eckart-Young-like result obtained by Kilmer et. al. states that $\mathcal{A}^{(q)}$ and $\mathcal{A}_{\boldsymbol{\rho}}$ are the 'best t/multi-rank $q, \boldsymbol{\rho}$' approximations of $\mathcal{A}$ respectively, where 'best' refers to entrywise squared error, i.e. the Frobenius norm of the error. In other words, $\mathcal{A}^{(qk)}$ and $\mathcal{A}_{\boldsymbol{\rho}}$ are the global minimizers of $\|\mathcal{A} - \mathcal{B}\|_F^2$ for $\mathcal{B}$ with t-rank q (respectively, multi-rank $\boldsymbol{\rho}$) under $\star_{\mathrm{M}}$ of the same dimensions as $\mathcal{A}$.

Let $\mathcal{A} = \mathcal{U} \star_{\mathrm{M}} \mathcal{S} \star_{\mathrm{M}} \mathcal{V}^{\mathbf{T}} \in \mathbb{R}^{m \times p \times n}$, we will use $j_1, \ldots, j_{np}$ and $i_1, \ldots, i_{np}$ to denote the indexes of the non-zeros of $\widehat{\mathcal{S}}$ ordered in decreasing order. That is

$$\hat{\sigma}_\ell := \hat{\sigma}_{j_\ell}^{(i_\ell)} \tag{4}$$

where $\hat{\sigma}_1 \geq \hat{\sigma}_2 \geq \cdots \geq \hat{\sigma}_{np}$.

In this work, we consider truncation with respect to the explicitly given implicit rank under $\star_{\mathrm{M}}$. For $q = 1, \ldots, pn$, the **explicit rank-q truncation** under $\star_{\mathrm{M}}$ of $\mathcal{A}$ is the tensor $\mathcal{A}_{\boldsymbol{\rho}}$ of multi-rank $\boldsymbol{\rho}$ under $\star_{\mathrm{M}}$ where

$$\boldsymbol{\rho}_i = \max\{j = 1, \ldots, p \mid (j, i) \in \{(j_1, j_1), \ldots, (j_q, i_q)\}\} \ . \tag{5}$$

In words, we keep the q top singular values of any frontal slice of $\widehat{\mathcal{S}}$, and zero out the rest. Note that the explicit rank-q truncation is not always uniquely defined by q since ties between singular values result in multiple possible choices for the multi-rank $\boldsymbol{\rho}$. However, all explicit-rank q truncations are equivalent in that they produce identical reconstruction errors. Indeed, let $\mathcal{A}_{\boldsymbol{\rho}}$ be a multi-rank $\boldsymbol{\rho}$ truncation of $\mathcal{A}$, implied by target explicit rank q, then $\|\mathcal{A} - \mathcal{A}_{\boldsymbol{\rho}}\|_F^2 = \sum_{\ell=q+1}^{p \cdot n} \hat{\sigma}_\ell^2$, meaning that the reconstruction error remains the same for any choice of multi-rank $\boldsymbol{\rho}$ in eq. (5). Furthermore, given the definition of explicit rank truncation, we get the following.

Claim B.1. *Suppose that* $\mathbf{M}$ *is a unitary matrix. Let* $\mathcal{A} \in \mathbb{R}^{m \times p \times n}$*, with a full* TSVDM $\mathcal{A} = \mathcal{U} \star_{\mathrm{M}} \mathcal{S} \star_{\mathrm{M}} \mathcal{V}^{\mathbf{T}}$*, and* $\boldsymbol{\rho} = [\boldsymbol{\rho}_1, \ldots, \boldsymbol{\rho}_n]$ *be the multi rank defined by explicit rank-q truncation of* $\mathcal{A}$ *in eq. (5). Let* $\mathcal{A}_{\boldsymbol{\rho}} = \mathcal{U}_{\boldsymbol{\rho}} \star_{\mathrm{M}} \mathcal{S}_{\boldsymbol{\rho}} \star_{\mathrm{M}} \mathcal{V}_{\boldsymbol{\rho}}^{\mathbf{T}} \in \mathbb{R}^{m \times p \times n}$*, the multi-rank* $\boldsymbol{\rho}$ *truncation of* $\mathcal{A}$*, then* $\mathcal{A}_{\boldsymbol{\rho}}$ *is the best implicit rank-q approximation of* $\mathcal{A}$*.*

Proof. Let $\boldsymbol{\varphi} = [\boldsymbol{\varphi}_1, \ldots, \boldsymbol{\varphi}_n]$ be a multi-rank such that $\sum_{i=1}^{n} \boldsymbol{\varphi}_i = q$. Then $\mathcal{A}_{\boldsymbol{\varphi}} = \mathcal{U}_{\boldsymbol{\varphi}} \star_{\mathrm{M}} \mathcal{S}_{\boldsymbol{\varphi}} \star_{\mathrm{M}} \mathcal{V}_{\boldsymbol{\varphi}}^{\mathbf{T}} \in \mathbb{R}^{m \times p \times n}$ is the best multi-rank $\boldsymbol{\varphi}$ approximation of $\mathcal{A}$. Let the tuples $(\tilde{j}_1, \tilde{i}_1), \ldots, (\tilde{j}_q, \tilde{i}_q)$ denote an ordering of the singular values $\{\hat{\sigma}_j^{(i)}\}$ for $i = 1, \ldots, n$ and $j = 1, \ldots, \boldsymbol{\varphi}_i$, such that $\hat{\sigma}_{\tilde{1}} \geq \hat{\sigma}_{\tilde{2}} \geq \cdots \geq \hat{\sigma}_{\tilde{q}}$ where $\hat{\sigma}_{\tilde{\ell}} = \hat{\sigma}_{\tilde{j}_\ell}^{(\tilde{i}_\ell)}$. Then, by construction of $\hat{\sigma}_\ell$ in eq. (4), we have that $\hat{\sigma}_\ell \geq \hat{\sigma}_{\tilde{\ell}}$ for all $\ell = 1, \ldots, q$, hence $\sum_{h=1}^{q} \hat{\sigma}_{\tilde{h}}^2 \leq \sum_{h=1}^{q} \hat{\sigma}_h^2$.

Now, we have that

$$\begin{aligned}
\|\mathcal{A} - \mathcal{A}_{\boldsymbol{\varphi}}\|_F^2 &= \sum_{i=1}^{n} \sum_{j=1}^{p} (\hat{\sigma}_j^{(i)})^2 - \sum_{i=1}^{n} \sum_{j=1}^{\boldsymbol{\varphi}_i} (\hat{\sigma}_j^{(i)})^2 \\
&= \sum_{h=1}^{pn} \hat{\sigma}_h^2 - \sum_{h=1}^{q} \hat{\sigma}_{\tilde{h}}^2 \\
&\geq \sum_{h=1}^{pn} \hat{\sigma}_h^2 - \sum_{h=1}^{q} \hat{\sigma}_h^2 \\
&= \|\mathcal{A} - \mathcal{A}_{\boldsymbol{\rho}}\|_F^2
\end{aligned}$$

Thus, we established that the best implicit rank q approximation of $\mathcal{A}$, is the tensor $\mathcal{A}_{\boldsymbol{\rho}}$ with multi-rank $\boldsymbol{\rho}$ implied by explicit rank q truncation of $\mathcal{A}$. ∎

We also note that for the explicit rank-q truncation of $\boldsymbol{\mathcal{A}}$, it holds that

$$\|\boldsymbol{\mathcal{A}}_{\boldsymbol{\rho}}\|_F^2 = \sum_{h=1}^{q} \hat{\sigma}_h^2 \tag{6}$$

where $\hat{\sigma}_h$ is defined by eq. (4).

B.3 A PCA-like tensor decomposition

The following construction was first presented in [8]. Short presentation of the objects we are concerned with will be followed by novel results regarding their algebraic and geometric properties. For elaborate definitions and discussion, see [8, 1]

Let $\boldsymbol{\mathcal{A}} = \boldsymbol{\mathcal{U}} \star_{\mathrm{M}} \boldsymbol{\mathcal{S}} \star_{\mathrm{M}} \boldsymbol{\mathcal{V}}^{\mathbf{T}} \in \mathbb{R}^{m\times p\times n}$ in mean deviation form (see Supporting Information D section), and consider the following expressions [8]:

$$\boldsymbol{\mathcal{Z}} = \boldsymbol{\mathcal{A}} \star_{\mathrm{M}} \boldsymbol{\mathcal{V}} \tag{7}$$

and

$$\boldsymbol{\mathcal{Z}}_{\boldsymbol{\rho}} := \boldsymbol{\mathcal{A}} \star_{\mathrm{M}} \boldsymbol{\mathcal{V}}_{\boldsymbol{\rho}} \tag{8}$$

where the tensor $\boldsymbol{\mathcal{V}}_{\boldsymbol{\rho}} \in \mathbb{R}^{p\times p\times n}$ is obtained from explicit rank q truncated TSVDM of $\boldsymbol{\mathcal{A}}$. The expressions in eq. (8) makes use in the TSVDM to form a construction of high resemblance to the truncated PCA shown in eq. (3). We also point that $\boldsymbol{\mathcal{V}}_{\boldsymbol{\rho}}$ is a member of particular family - it is a pseudo $\star_{\mathrm{M}}$-orthogonal tensor of implicit rank q.

Let $\boldsymbol{\mathcal{B}} \in \mathbb{R}^{p\times p\times n}$ be a pseudo $\star_{\mathrm{M}}$-orthogonal tensor of implicit rank q, and notice that $(\boldsymbol{\mathcal{B}}^{\mathbf{T}} \star_{\mathrm{M}} \boldsymbol{\mathcal{B}}) \times_3 \mathbf{M}$ is an f-diagonal tensor with diagonals consists of zeros and ones, and for which the number of non-zero entries is q. Define

$$\boldsymbol{\mathcal{Y}} = \boldsymbol{\mathcal{A}} \star_{\mathrm{M}} \boldsymbol{\mathcal{B}} \tag{9}$$

In Property A.1 the formulation is concerned with maximizing (or minimizing) the variance component of a random variable $\mathbf{y}$, that is quantified using the trace of the variance-covariance matrix of that random variable. The objective of maximizing (minimizing) the sample variance of $\mathbf{Y}$, that is proportional to $\mathrm{Tr}(\mathbf{B}^{\mathbf{T}}\mathbf{A}^{\mathbf{T}}\mathbf{A}\mathbf{B})$, which, by definition of the Frobenius norm, results in $\|\mathbf{A}\mathbf{B}\|_F^2$. In absence of analog definitions for variance of a tensor valued random variable and the trace of a tensor, we resort to discuss an algebraically similar trait.

Define the sample variance-covariance tensor [8] for $\boldsymbol{\mathcal{Y}}$ as

$$\boldsymbol{\mathcal{E}}_{\boldsymbol{\mathcal{Y}}} := \boldsymbol{\mathcal{B}}^{\mathbf{T}} \star_{\mathrm{M}} \boldsymbol{\mathcal{E}} \star_{\mathrm{M}} \boldsymbol{\mathcal{B}} \tag{10}$$

where $\boldsymbol{\mathcal{E}} = (m-1)^{-1}\boldsymbol{\mathcal{A}}^{\mathbf{T}} \star_{\mathrm{M}} \boldsymbol{\mathcal{A}} \in \mathbb{R}^{p\times p\times n}$ is the sample variance-covariance tensor for $\boldsymbol{\mathcal{A}}$ (and $\boldsymbol{\mathcal{Z}}$).

Property B.2. *Suppose that* $\mathbf{M}$ *is a unitary matrix. Let* $\boldsymbol{\mathcal{B}} \in \mathbb{R}^{p\times p\times n}$ *a* pseudo $\star_{\mathrm{M}}$-orthogonal *tensor of implicit rank* q*. Then the quantity* $\|\boldsymbol{\mathcal{A}} \star_{\mathrm{M}} \boldsymbol{\mathcal{B}}\|_F^2$ *is maximized when* $\boldsymbol{\mathcal{B}} = \boldsymbol{\mathcal{V}}_{\boldsymbol{\rho}}$*, where* $\boldsymbol{\mathcal{A}}_{\boldsymbol{\rho}} = \boldsymbol{\mathcal{U}}_{\boldsymbol{\rho}} \star_{\mathrm{M}} \boldsymbol{\mathcal{S}}_{\boldsymbol{\rho}} \star_{\mathrm{M}} \boldsymbol{\mathcal{V}}_{\boldsymbol{\rho}}^{\mathbf{T}}$ *is the explicit rank* q *truncation of* $\boldsymbol{\mathcal{A}}$*.*

Proof of Property B.2. We have that $\|\boldsymbol{\mathcal{A}} \star_{\mathrm{M}} \boldsymbol{\mathcal{B}}\|_F^2 = \|\widehat{\boldsymbol{\mathcal{A}}} \vartriangle \widehat{\boldsymbol{\mathcal{B}}}\|_F^2 = \sum_{i=1}^{n} \mathrm{Tr}(\widehat{\boldsymbol{\mathcal{B}}}^{\mathbf{T}}_{:,:,i}\widehat{\boldsymbol{\mathcal{A}}}^{\mathbf{T}}_{:,:,i}\widehat{\boldsymbol{\mathcal{A}}}_{:,:,i}\widehat{\boldsymbol{\mathcal{B}}}_{:,:,i})$. Recall that $\boldsymbol{\mathcal{A}} = \boldsymbol{\mathcal{U}} \star_{\mathrm{M}} \boldsymbol{\mathcal{S}} \star_{\mathrm{M}} \boldsymbol{\mathcal{V}}^{\mathbf{T}}$, and write $\boldsymbol{\mathcal{B}} = \boldsymbol{\mathcal{V}} \star_{\mathrm{M}} \boldsymbol{\mathcal{C}}$ where $\boldsymbol{\mathcal{C}} = \boldsymbol{\mathcal{V}}^{\mathbf{T}} \star_{\mathrm{M}} \boldsymbol{\mathcal{B}}$. Note that $\boldsymbol{\mathcal{C}}$ is also an implicit rank q pseudo $\star_{\mathrm{M}}$-orthogonal tensor. So, $\|\boldsymbol{\mathcal{A}} \star_{\mathrm{M}} \boldsymbol{\mathcal{B}}\|_F^2 = \sum_{i=1}^{n} \mathrm{Tr}(\widehat{\boldsymbol{\mathcal{C}}}^{\mathbf{T}}_{:,:,i}\widehat{\boldsymbol{\mathcal{S}}}^{\mathbf{T}}_{:,:,i}\widehat{\boldsymbol{\mathcal{S}}}_{:,:,i}\widehat{\boldsymbol{\mathcal{C}}}_{:,:,i}) = \|\boldsymbol{\mathcal{S}} \star_{\mathrm{M}} \boldsymbol{\mathcal{C}}\|_F^2$. Thus $\|\boldsymbol{\mathcal{A}} \star_{\mathrm{M}} \boldsymbol{\mathcal{B}}\|_F^2 = \sum_{i=1}^{n}\sum_{j=1}^{p}(\hat{\sigma}_j^{(i)})^2 c_{j,i}$ where $c_{j,i} = \widehat{\boldsymbol{\mathcal{C}}}_{j,:,i}\widehat{\boldsymbol{\mathcal{C}}}^{\mathbf{T}}_{j,:,i}$, which may be re-ordered according to eq. (4) to obtain

$$\|\boldsymbol{\mathcal{A}} \star_{\mathrm{M}} \boldsymbol{\mathcal{B}}\|_F^2 = \sum_{h=1}^{pn} \hat{\sigma}_h^2 c_{j_h,i_h}$$

Since $0 \le c_{j_h,i_h} \le 1$ and $\|\boldsymbol{\mathcal{C}}\|_F^2 = \sum_{j=1}^{p} c_{j,i} = q$, we have that $\sum_{h=1}^{pn}\hat{\sigma}_h^2 c_{j_h,i_h} \le \sum_{h=1}^{q}\hat{\sigma}_h^2$. Hence, $\|\boldsymbol{\mathcal{A}} \star_{\mathrm{M}} \boldsymbol{\mathcal{B}}\|_F^2 \le \sum_{h=1}^{q}\hat{\sigma}_h^2$, which according to eq. (6), equals to $\|\boldsymbol{\mathcal{A}}_{\boldsymbol{\rho}}\|_F^2$ where $\boldsymbol{\rho}$ is the multi-rank implied by explicit rank q truncation of $\boldsymbol{\mathcal{A}}$ (See eq. (5)).

Note that taking $\boldsymbol{\mathcal{B}} = \boldsymbol{\mathcal{V}}_{\boldsymbol{\rho}}$ yield $c_{j,i} = ((\widehat{\boldsymbol{\mathcal{V}}}_{:,j,i})^{\mathbf{T}}(\widehat{\boldsymbol{\mathcal{V}}}_{\boldsymbol{\rho}})_{:,:,i})((\widehat{\boldsymbol{\mathcal{V}}}_{:,j,i})^{\mathbf{T}}(\widehat{\boldsymbol{\mathcal{V}}}_{\boldsymbol{\rho}})_{:,:,i})^{\mathbf{T}}$, that in turn results in

$$c_{j,i} = \begin{cases} 1 & \exists h \le q \text{ s.t. } (j_h, i_h) = (j,i) \\ 0 & \text{otherwise} \end{cases}$$

thus, the upper bound is achieved for $\|\boldsymbol{\mathcal{A}} \star_{\mathrm{M}} \boldsymbol{\mathcal{V}}_{\boldsymbol{\rho}}\|_F^2 = \sum_{h=1}^{q}\hat{\sigma}_h^2$.

□

The tensor equivalent result for Property A.3 says the minimizer of the distortion under pseudo-orthogonality constraints is again $\boldsymbol{\mathcal{V}}_{\boldsymbol{\rho}}$.

Property B.3. *Suppose that* $\mathbf{M}$ *is a unitary matrix. Let* $\boldsymbol{\mathcal{A}} \in \mathbb{R}^{m\times p\times n}$ *in mean deviation form and define* $\boldsymbol{\mathcal{Y}} = \boldsymbol{\mathcal{A}} \star_{\mathbf{M}} \boldsymbol{\mathcal{B}} \in \mathbb{R}^{m\times p\times n}$ *where* $\boldsymbol{\mathcal{B}} \in \mathbb{R}^{p\times p\times n}$ *is a* pseudo $\star_{\mathbf{M}}$-orthogonal *tensor of implicit rank* q*. Then the distortion formed by* $\boldsymbol{\mathcal{B}}$*, measured as* $\|\boldsymbol{\mathcal{Y}} \star_{\mathbf{M}} \boldsymbol{\mathcal{Y}}^{\mathbf{T}} - \boldsymbol{\mathcal{A}} \star_{\mathbf{M}} \boldsymbol{\mathcal{A}}^{\mathbf{T}}\|_F^2$*, is minimized when* $\boldsymbol{\mathcal{B}} = \boldsymbol{\mathcal{V}}_{\boldsymbol{\rho}}$*.*

Proof of Property B.3. Note that $\widehat{\boldsymbol{\mathcal{A}}}_{:,:,i}\widehat{\boldsymbol{\mathcal{A}}}^{\mathbf{T}}_{:,:,i}$ is symmetric positive semidefinite, thus, $\widehat{\boldsymbol{\mathcal{A}}}_{:,:,i}\widehat{\boldsymbol{\mathcal{A}}}^{\mathbf{T}}_{:,:,i} = \sum_{j=1}^{\boldsymbol{\theta}_i}(\hat{\sigma}_j^{(i)})^2\mathbf{u}_j^i(\mathbf{u}_j^i)^{\mathbf{T}}$ where $\{\mathbf{u}_j^i\}_{j=1}^{\boldsymbol{\theta}_i}$ are unit normalized orthogonal vectors, $(\hat{\sigma}_1^{(i)})^2 \geq (\hat{\sigma}_2^{(i)})^2 \geq \cdots \geq (\hat{\sigma}_{\boldsymbol{\theta}_i}^{(i)})^2$ are the eigenvalues of $\widehat{\boldsymbol{\mathcal{A}}}_{:,:,i}\widehat{\boldsymbol{\mathcal{A}}}^{\mathbf{T}}_{:,:,i}$, and $\boldsymbol{\theta} \coloneqq [\boldsymbol{\theta}_1, \ldots, \boldsymbol{\theta}_n]$ is the multi-rank of $\boldsymbol{\mathcal{A}}$ under $\star_{\mathbf{M}}$. Similarly, we have that $\widehat{\boldsymbol{\mathcal{Y}}}_{:,:,i}\widehat{\boldsymbol{\mathcal{Y}}}^{\mathbf{T}}_{:,:,i} = \sum_{j=1}^{\boldsymbol{\varphi}_i}\mu_j^i\mathbf{p}_j^i(\mathbf{p}_j^i)^{\mathbf{T}}$ where $\{\mathbf{p}_j^i\}_{j=1}^{\boldsymbol{\varphi}_i}$ are unit normalized orthogonal vectors, $\mu_1^i \geq \mu_2^i \geq \cdots \geq \mu_{\boldsymbol{\varphi}_i}^i$ are the eigenvalues of $\widehat{\boldsymbol{\mathcal{Y}}}_{:,:,i}\widehat{\boldsymbol{\mathcal{Y}}}^{\mathbf{T}}_{:,:,i}$, and $\boldsymbol{\varphi}_i$ denote the rank of $\widehat{\boldsymbol{\mathcal{Y}}}_{:,:,i}\widehat{\boldsymbol{\mathcal{Y}}}^{\mathbf{T}}_{:,:,i}$.

Note that $\widehat{\boldsymbol{\mathcal{Y}}}_{:,:,i}\widehat{\boldsymbol{\mathcal{Y}}}^{\mathbf{T}}_{:,:,i} = \sum_{j=1}^{\boldsymbol{\theta}_i}\mu_j^i\mathbf{u}_j^i\widehat{\boldsymbol{\mathcal{B}}}_{:,:,i}\widehat{\boldsymbol{\mathcal{B}}}^{\mathbf{T}}_{:,:,i}(\mathbf{u}_j^i)^{\mathbf{T}}$, therefore, it must hold that $\boldsymbol{\varphi}_i \leq \boldsymbol{\theta}_i$ for all $i = 1, \ldots, n$, meaning that $\sum_{j=1}^{\boldsymbol{\varphi}_i}(\hat{\sigma}_j^{(i)})^2\mathbf{u}_j^i(\mathbf{u}_j^i)^{\mathbf{T}}$ is the best rank $\boldsymbol{\varphi}_i$ approximation of $\widehat{\boldsymbol{\mathcal{A}}}_{:,:,i}\widehat{\boldsymbol{\mathcal{A}}}^{\mathbf{T}}_{:,:,i}$. As a result, we have $\|\widehat{\boldsymbol{\mathcal{Y}}}_{:,:,i}\widehat{\boldsymbol{\mathcal{Y}}}^{\mathbf{T}}_{:,:,i} - \widehat{\boldsymbol{\mathcal{A}}}_{:,:,i}\widehat{\boldsymbol{\mathcal{A}}}^{\mathbf{T}}_{:,:,i}\|_F^2 \geq \sum_{j=\boldsymbol{\varphi}_i+1}^{\boldsymbol{\theta}_i}(\hat{\sigma}_j^{(i)})^4$ for all i. Moreover, since $\boldsymbol{\mathcal{B}}$ is of implicit rank q, it holds that $\sum_{i=1}^{n}\boldsymbol{\varphi}_i \leq q$. Combining the last two, we have

$$\begin{aligned}\|\boldsymbol{\mathcal{Y}} \star_{\mathbf{M}} \boldsymbol{\mathcal{Y}}^{\mathbf{T}} - \boldsymbol{\mathcal{A}} \star_{\mathbf{M}} \boldsymbol{\mathcal{A}}^{\mathbf{T}}\|_F^2 &\geq \sum_{i=1}^{n}\sum_{j=1}^{\boldsymbol{\theta}_i}(\hat{\sigma}_j^{(i)})^4 - \sum_{i=1}^{n}\sum_{j=1}^{\boldsymbol{\varphi}_i}(\hat{\sigma}_j^{(i)})^4 \\ &\geq \sum_{h=1}^{np}(\hat{\sigma}_h)^4 - \sum_{h=1}^{q}(\hat{\sigma}_h)^4\end{aligned}$$

where $\hat{\sigma}_h \coloneqq \hat{\sigma}_{j_h,i_h}$ (See eq. (4)). Simple calculations reveals that setting $\boldsymbol{\mathcal{B}} = \boldsymbol{\mathcal{V}}_{\boldsymbol{\rho}}$ results in exactly

$$\|\boldsymbol{\mathcal{A}} \star_{\mathbf{M}} \boldsymbol{\mathcal{V}}_{\boldsymbol{\rho}} \star_{\mathbf{M}} \boldsymbol{\mathcal{V}}_{\boldsymbol{\rho}}^{\mathbf{T}} \star_{\mathbf{M}} \boldsymbol{\mathcal{A}}^{\mathbf{T}} - \boldsymbol{\mathcal{A}} \star_{\mathbf{M}} \boldsymbol{\mathcal{A}}^{\mathbf{T}}\|_F^2 = \sum_{h=1}^{np}(\hat{\sigma}_h)^4 - \sum_{h=1}^{q}(\hat{\sigma}_h)^4$$

so we conclude that $\boldsymbol{\mathcal{V}}_{\boldsymbol{\rho}}$ is a global minimizer of the distortion as it reaches the global lower bound. □

B.4 Explicit rank truncated tensors in vector representation

Having established optimality properties with respect to variance and distortion for explicit rank truncated tensors (Properties B.2 and B.3), we turn to do the same for vector representation of these truncations. The vector form of truncated tensors is obtained by mode-1 unfolding. Let $\boldsymbol{\mathcal{X}} \in \mathbb{R}^{1\times p\times n}$, we define $\mathrm{vec}(\boldsymbol{\mathcal{X}}) \in \mathbb{R}^{pn}$ as

$$\mathrm{vec}(\boldsymbol{\mathcal{X}}) = [\boldsymbol{\mathcal{X}}_{1,1,1}, \boldsymbol{\mathcal{X}}_{1,2,1}, \ldots, \boldsymbol{\mathcal{X}}_{1,p,1}, \boldsymbol{\mathcal{X}}_{1,1,2}, \boldsymbol{\mathcal{X}}_{1,2,2}, \ldots, \boldsymbol{\mathcal{X}}_{1,p,n}] \ .$$

For tensors $\boldsymbol{\mathcal{X}} \in \mathbb{R}^{m\times p\times n}$, we slightly abuse the above notation by letting $\mathrm{vec}(\boldsymbol{\mathcal{X}}) \in \mathbb{R}^{m\times np}$ denote the matrix whose ℓ^{th} row is $\mathrm{vec}(\boldsymbol{\mathcal{X}}_{\ell,:,:})$ (so, even if $m = 1$ we will view $\mathrm{vec}(\boldsymbol{\mathcal{X}})$ as a row vector).

Consider the family $\mathcal{Q}$ of tensor-vector mappings $\mathbb{R}^{m\times p\times n}$ to $\mathbb{R}^{m\times pn}$ whose members are of the form

$$Q_{\boldsymbol{\mathcal{B}}}(\boldsymbol{\mathcal{X}}) = \mathrm{vec}((\boldsymbol{\mathcal{X}} \star_{\mathbf{M}} \boldsymbol{\mathcal{B}}) \times_3 \mathbf{M})$$

where $\boldsymbol{\mathcal{B}} \in \mathbb{R}^{p\times p\times n}$ is a pseudo $\star_{\mathbf{M}}$-orthogonal tensor. We define the **rank** of a member $Q_{\boldsymbol{\mathcal{B}}}$ in $\mathcal{Q}$ as the implicit rank of $\boldsymbol{\mathcal{B}}$.

In analogy to Supporting Information A and B.3 we present the following properties.

Property B.4. *Suppose that* $\mathbf{M}$ *is a unitary matrix. Let* $\boldsymbol{\mathcal{A}} \in \mathbb{R}^{m\times p\times n}$ *be in mean deviation form and* $\mathbf{Y} \coloneqq Q_{\boldsymbol{\mathcal{B}}}(\boldsymbol{\mathcal{A}}) \in \mathbb{R}^{m\times np}$ *for some* pseudo $\star_{\mathbf{M}}$-orthogonal $\boldsymbol{\mathcal{B}}$*. As in Supporting Information A, we define* $\mathbf{S}_{\mathbf{Y}} \coloneqq (m-1)^{-1}\mathbf{Y}^{\mathbf{T}}\mathbf{Y}$ *the sample variance-covariance matrix of* $\mathbf{Y}$*. Then, the rank* q *member of* $\mathcal{Q}$ *for which the variance component* $(m-1)^{-1}\,\mathrm{Tr}(\mathbf{Y}^{\mathbf{T}}\mathbf{Y})$ *is maximized, is given by* $Q_{\boldsymbol{\mathcal{V}}_{\boldsymbol{\rho}}}$ *where* $\boldsymbol{\mathcal{A}}_{\boldsymbol{\rho}} = \boldsymbol{\mathcal{U}}_{\boldsymbol{\rho}} \star_{\mathbf{M}} \boldsymbol{\mathcal{S}}_{\boldsymbol{\rho}} \star_{\mathbf{M}} \boldsymbol{\mathcal{V}}_{\boldsymbol{\rho}}^{\mathbf{T}}$ *is the multi-rank* $\boldsymbol{\rho}$ *truncation* $\boldsymbol{\mathcal{A}}$ *under* $\star_{\mathbf{M}}$ *implied by explicit rank* q *truncation of* $\boldsymbol{\mathcal{A}}$*.*

Proof of Property B.4. Note that

$$\begin{aligned}
\mathrm{Tr}(\mathbf{S}_{\mathbf{Y}}) &\propto \mathrm{Tr}(\mathbf{Y}\mathbf{Y}^{\mathbf{T}}) \\
&= \mathrm{Tr}(\sum_{h=1}^{np} \mathbf{Y}_{:,h}(\mathbf{Y}_{:,h})^{\mathbf{T}}) \\
&= \sum_{h=1}^{np} \mathrm{Tr}(\mathbf{Y}_{:,h}(\mathbf{Y}_{:,h})^{\mathbf{T}}) \\
&= \sum_{i=1}^{n}\sum_{j=1}^{p} \mathrm{Tr}(\widehat{\mathcal{Y}}_{:,j,i}(\widehat{\mathcal{Y}}_{:,j,i})^{\mathbf{T}}) \\
&= \sum_{i=1}^{n}\sum_{j=1}^{p} (\widehat{\mathcal{Y}}_{:,j,i})^{\mathbf{T}}\widehat{\mathcal{Y}}_{:,j,i} \\
&= \sum_{i=1}^{n} \|\widehat{\mathcal{Y}}_{:,:,i}\|_F^2 = \|\mathcal{Y}\|_F^2
\end{aligned}$$

where $\mathcal{Y} = \mathcal{A} \star_{\mathrm{M}} \mathcal{B}$. So we conclude that $\mathrm{Tr}(\mathbf{S}_{\mathbf{Y}}) = (m-1)^{-1}\|\mathcal{A} \star_{\mathrm{M}} \mathcal{B}\|_F^2$ Now, by Property B.2, we have that the implicit rank q for which this quantity is maximal is $\mathcal{V}_{\boldsymbol{\rho}}$. □

Property B.4 provides a variance maximization result for flattened explicit rank q truncations that is similar to Properties A.1 and B.2 which state variance maximization for traditional rank q truncations of matrices (PCA) and explicit rank-q truncations of tensors respectively.

We proceed with presenting a slightly modified version of Properties A.3 and B.3. Recall that Properties A.3 and B.3 discuss the minimization of the Frobenius norm of the difference between configuration matrices (or tensors) of the truncated and of the complete representation ;

$$\|\mathbf{Y}\mathbf{Y}^{\mathbf{T}} - \mathbf{A}\mathbf{A}^{\mathbf{T}}\|_F^2, \qquad\qquad \|\mathcal{Y} \star_{\mathrm{M}} \mathcal{Y}^{\mathbf{T}} - \mathcal{A} \star_{\mathrm{M}} \mathcal{A}^{\mathbf{T}}\|_F^2$$

Formulating analog property for flattened rank q truncated representations, requires a definition of the complete flattened representation of a tensor. Given $\mathcal{A} \in \mathbb{R}^{m\times p\times n}$ in mean deviation form, we let

$$\mathbf{A} := Q_{\mathcal{V}}(\mathcal{A}) = \mathrm{vec}(\widehat{\mathcal{Z}}) \tag{11}$$

denote the complete flattened representation of $\mathcal{A}$, where $\mathcal{A} = \mathcal{U} \star_{\mathrm{M}} \mathcal{S} \star_{\mathrm{M}} \mathcal{V}^{\mathbf{T}} = \mathcal{Z} \star_{\mathrm{M}} \mathcal{V}^{\mathbf{T}}$. We argue that the definition of the complete flattened representation stated above is rather natural since it holds all information regarding the original tensor $\mathcal{A}$, and reconstructing $\mathcal{A}$ from $\mathbf{A}$ is possible by applying an inverse vec operation to $\mathbf{A}$, followed by a $\times_3\mathbf{M}^{-1}$ tensor-matrix product, and a right $\star_{\mathrm{M}}$ product with $\mathcal{V}^{\mathbf{T}}$. Additional argument in favor of the choice in eq. (11), is that the sample variance component in $\mathbf{S}_{\mathbf{A}}$ is identical to that of the original tensor, as could be deduced from the proof of Property B.4.

Therefore, we will use $\mathbf{A}$ (eq. (11)) as the reference point for quantifying the distortion in configuration of flattened truncated rank representations of $\mathcal{A}$. Note that in contrast to the fact that the expressions $\|\mathcal{Y}\|_F^2$ and $\|\mathbf{Y}\|_F^2$ are identical (a fact that was used to show Property B.4), the quantities $\|\mathcal{Y} \star_{\mathrm{M}} \mathcal{Y}^{\mathbf{T}}\|_F^2$ and $\|\mathbf{Y}\mathbf{Y}^{\mathbf{T}}\|_F^2$ are not equal in general. This forces a slight modification in the definition of the concept of distortion itself. Instead of using the traditional formulation $\|\mathbf{Y}\mathbf{Y}^{\mathbf{T}} - \mathbf{A}\mathbf{A}^{\mathbf{T}}\|_F^2$ as in [7], we replace the Frobenius norm with the nuclear norm. Given a matrix $\mathbf{X}$, the nuclear norm of $\mathbf{X}$, denoted by $\|\mathbf{X}\|_*$, is defined as the sum of $\mathbf{X}$'s singular values. In the special case where $\mathbf{X}$ is a positive semi-definite matrix (a case which we briefly describe as $\mathbf{X} \succeq 0$), we have that $\|\mathbf{X}\|_* = \mathrm{Tr}(\mathbf{X})$. We now show the following.

Property B.5. *Suppose that* $\mathbf{M}$ *is a unitary matrix. Let* $\mathcal{A} \in \mathbb{R}^{m\times p\times n}$ *be in mean deviation form,* $\mathbf{Y} := Q_{\mathcal{B}}(\mathcal{A}) \in \mathbb{R}^{m\times np}$ *and* $\mathbf{A} \in \mathbb{R}^{m\times pn}$ *defined by eq. (11). Then, the rank* q *member of* $\mathcal{Q}$ *for which the distortion* $\|\mathbf{Y}\mathbf{Y}^{\mathbf{T}} - \mathbf{A}\mathbf{A}^{\mathbf{T}}\|_*$ *is minimized, is given by* $Q_{\mathcal{V}_{\boldsymbol{\rho}}}$ *where* $\mathcal{A}_{\boldsymbol{\rho}} = \mathcal{U}_{\boldsymbol{\rho}} \star_{\mathrm{M}} \mathcal{S}_{\boldsymbol{\rho}} \star_{\mathrm{M}} \mathcal{V}_{\boldsymbol{\rho}}^{\mathbf{T}}$ *is the multi-rank* $\boldsymbol{\rho}$ *truncation* $\mathcal{A}$ *under* $\star_{\mathrm{M}}$ *implied by explicit rank* q *truncation of* $\mathcal{A}$*.*

Proof of Property B.5. Begin by noting that for any ℓ and k between 1 and m, we have

$$\begin{aligned}
[\mathbf{Y}\mathbf{Y}^{\mathbf{T}}]_{\ell,k} &= \mathrm{vec}(\widehat{\mathcal{Y}}_{\ell,:,:})\,\mathrm{vec}(\widehat{\mathcal{Y}}_{k,:,:})^{\mathbf{T}} \\
&= \sum_{i=1}^{n} \widehat{\mathcal{Y}}_{\ell,:,i}\widehat{\mathcal{Y}}_{k,:,i}^{\mathbf{T}} \\
&= (\sum_{i=1}^{n} \widehat{\mathcal{Y}}_{:,:,i}\widehat{\mathcal{Y}}_{:,:,i}^{\mathbf{T}})_{\ell,k}
\end{aligned}$$

and similarly, we get $[\mathbf{A}\mathbf{A}^\mathbf{T}]_{\ell,k} = (\sum_{i=1}^n \widehat{\mathcal{Z}}_{:,:,i}\widehat{\mathcal{Z}}^\mathbf{T}_{:,:,i})_{\ell,k}$, thus, $\mathbf{Y}\mathbf{Y}^\mathbf{T} - \mathbf{A}\mathbf{A}^\mathbf{T} = \sum_{i=1}^n \widehat{\mathcal{Y}}_{:,:,i}\widehat{\mathcal{Y}}^\mathbf{T}_{:,:,i} - \widehat{\mathcal{Z}}_{:,:,i}\widehat{\mathcal{Z}}^\mathbf{T}_{:,:,i}$. We write $\mathcal{Q} = \mathcal{V}^\mathbf{T} \star_\mathrm{M} \mathcal{B}$, and note that $\mathcal{Q}$ is a pseudo $\star_\mathrm{M}$ -orthogonal tensor of implicit rank q, then

$$\widehat{\mathcal{Y}}_{:,:,i}\widehat{\mathcal{Y}}^\mathbf{T}_{:,:,i} - \widehat{\mathcal{Z}}_{:,:,i}\widehat{\mathcal{Z}}^\mathbf{T}_{:,:,i} = \widehat{\mathcal{Z}}_{:,:,i}(\widehat{\mathcal{Q}}_{:,:,i}\widehat{\mathcal{Q}}^\mathbf{T}_{:,:,i} - \mathbf{I})\widehat{\mathcal{Z}}^\mathbf{T}_{:,:,i}$$

where $\widehat{\mathcal{Q}}_{:,:,i}\widehat{\mathcal{Q}}^\mathbf{T}_{:,:,i} - \mathbf{I}$ is a $p \times p$ symmetric matrix. Since $\mathbf{I} - \widehat{\mathcal{Q}}_{:,:,i}\widehat{\mathcal{Q}}^\mathbf{T}_{:,:,i} \succeq 0$ (it is a projection matrix), we have that $\mathbf{A}\mathbf{A}^\mathbf{T} - \mathbf{Y}\mathbf{Y}^\mathbf{T} \succeq 0$, and hence

$$\|\mathbf{A}\mathbf{A}^\mathbf{T} - \mathbf{Y}\mathbf{Y}^\mathbf{T}\|_* = \mathrm{Tr}(\mathbf{A}\mathbf{A}^\mathbf{T} - \mathbf{Y}\mathbf{Y}^\mathbf{T}) \ .$$

Note that

$$\begin{aligned}\mathrm{Tr}(\mathbf{A}\mathbf{A}^\mathbf{T} - \mathbf{Y}\mathbf{Y}^\mathbf{T}) &= \mathrm{Tr}(\sum_{i=1}^n \widehat{\mathcal{Z}}_{:,:,i}\widehat{\mathcal{Z}}^\mathbf{T}_{:,:,i} - \widehat{\mathcal{Y}}_{:,:,i}\widehat{\mathcal{Y}}^\mathbf{T}_{:,:,i}) \\ &= \|\mathcal{Z}\|_F^2 - \|\mathcal{Y}\|_F^2\end{aligned}$$

a quantity that that is minimized when $\|\mathcal{Y}\|_F^2$ is maximized. Since $\|\mathcal{Y}\|_F^2$ is proportional to the sample variance component of $\mathcal{Y}$, according to the proof of Property B.2, $\|\mathbf{A}\mathbf{A}^\mathbf{T} - \mathbf{Y}\mathbf{Y}^\mathbf{T}\|_*^2$ is minimized when $\mathbf{Y} = Q_{\mathcal{V}_\rho}(\mathcal{A})$, that is, $\mathcal{B} = \mathcal{V}_\rho$.

□

C TCAM

A closer inspection of $\mathcal{V}_\rho$ reveals that the flattened rank q truncated representation $Q_{\mathcal{V}_\rho}$, which was shown in Supporting Information B.4 to have optimality guarantees with respect to distortion and variance, can be considerably compressed. Consider the ith frontal face of $\widehat{\mathcal{V}}_\rho$, then we have that

$$\|(\widehat{\mathcal{V}}_\rho)_{:,j,i}\|_F^2 = \begin{cases} 1 & \exists h \leq q \ \text{ s.t. } \ (j_h, i_h) = (j, i) \\ 0 & \text{otherwise} \end{cases}$$

where we used the ordering of frontal and lateral indices defined in eq. (4). Thus, for any tensor $\mathcal{X} \in \mathbb{R}^{1\times p\times n}$ we have that $\widehat{\mathcal{X}}_{:,:,i}(\widehat{\mathcal{V}}_\rho)_{:,:,i}$ can obtain nonzero values only for indices $j = 1, \ldots, p$ such that $(j, i) = (j_h, i_h)$ for some $h \leq q$, essentially making $Q_{\mathcal{V}_\rho}$ a mapping from $\mathbb{R}^{1\times p\times n}$ to $\mathbb{R}^q$.

Given $\mathcal{A} \in \mathbb{R}^{m\times p\times n}$ with explicit rank q truncation $\mathcal{A}_\rho = \mathcal{U}_\rho \star_\mathrm{M} \mathcal{S}_\rho \star_\mathrm{M} \mathcal{V}_\rho^\mathbf{T}$, we let $Q : \mathbb{R}^{1\times p\times n} \to \mathbb{R}^q$ denote the compact representation of $Q_{\mathcal{V}_\rho}$;

$$Q(\mathcal{X}) = [x_1, x_2, ..., x_q]$$

where $x_h = [(\mathcal{X} \star_\mathrm{M} \mathcal{V}_\rho) \times_3 \mathbf{M}]_{:,j_h,i_h}$.

For any $\mathcal{X} \in \mathbb{R}^{k\times p\times n}$, let $\mathbf{X} := Q_{\mathcal{V}_\rho}(\mathcal{X})$ and $\tilde{\mathbf{X}} := Q(\mathcal{X})$. Clearly, $\mathrm{Tr}(\tilde{\mathbf{X}}^\mathbf{T}\tilde{\mathbf{X}}) = \mathrm{Tr}(\mathbf{X}^\mathbf{T}\mathbf{X})$ and also $\tilde{\mathbf{X}}\tilde{\mathbf{X}}^\mathbf{T} = \mathbf{X}\mathbf{X}^\mathbf{T}$. This gives rise to the following:

Definition C.1 (TCAM). *Let $\mathcal{A} \in \mathbb{R}^{m\times p\times n}$ in mean deviation form. The TCAM of $\mathcal{A}$ consists of the following:*

1. *The TCAM scores, given by the tensor-to-vector mapping Q specified above. Note that given a new sample $\mathcal{X} \in \mathbb{R}^{1\times p\times n}$, the TCAM scores of $\mathcal{X}$ may be easily obtained by applying Q to $\mathcal{X}$.*

2. *The TCAM loadings (or coefficients) matrix $\mathbf{V} \in \mathbb{R}^{np\times p}$ with entries $\mathbf{V}_{h,j} = \widehat{\mathcal{V}}_{j_h,j,i_h}$, where the index h corresponds to the ordering of the singular values in eq. (4).*

The TCAM may be truncated at any target number of factors q by taking the first q rows for the scores matrix, and the first q rows in the coefficients matrix.

The scores of the q truncated TCAM enjoy the same properties as $Q_{\mathcal{V}_\rho}(\mathcal{A})$, hence, these scores make variance maximizing vector representation of samples in $\mathcal{A}$ (Property B.4) while minimizing the distortion with respect to the nuclear norm (Property B.5).

D Data pre-processing

Baseline normalization. For experiment with timepoints $t_1, \ldots, t_n$, where $t_1, \ldots, t_j$ are considered the baseline samples. Let $s_{\ell 1}, \ldots, s_{\ell n}$ denote the samples collected from subject ℓ. Assuming all data are non-negative (as in count data or relative abundance), the log-folds from baseline (LFB) transformation of the data $\hat{s}_{\ell 1}, \ldots, \hat{s}_{\ell n}$ is defined by $\hat{s}_{\ell k} := \log_2(s_{\ell k}/\bar{s}_\ell)$ where $\bar{s}_\ell$ is the mean of subject ℓ's baseline samples: $s_{\ell 1}, \ldots, s_{\ell j}$. In cases data are normalized, the deviation from baseline (DFB) transformed data $\hat{s}_{\ell 1}, \ldots, \hat{s}_{\ell n}$ is obtained by $\hat{s}_{\ell k} := s_{\ell k} - \bar{s}_\ell$.

Post antibiotics reconstitution. Shotgun metagenomics sequencing data of stool samples was downloaded from ENA (project accession number: PRJEB28097). QC filtration and read trimming were done using fastp, followed by removal of reads mapped to human genome by bowtie2 mapper. MetaPhlan3 was used for taxonomic assignment of the reads. For the analysis, we included the timepoints with missing samples of no more than 2 subjects, allowing for single day deviation in any direction. Subjects without at least one sample in each phase of the experiment (baseline, antibiotics, intervention) were excluded. Relative abundance values were capped at 10^{-6} and features with maximal values less than 10^{-6} were omitted. For the analysis using TCAM, data from each participant were LFBtransformed. PERMANOVA was computed using truncated distance matrices reconstructed using the minimal number of components (either TCAM or PCA) such that the truncation accounts for at least 20% of the total variation in the data. Feature selection for univariate time-series analysis was done by taking the 0.75 quantile of loadings norm computed for the factors demonstrating significant univariate difference (ANOVA). Univariate time-series analysis was performed using lmer.

ECAM dataset. 16S rDNA sequencing data and metadata for [9] were obtained from a repository set up by Martino et. al. [10]. Relative abundance values were capped at 10^{-3}, i.e. features with maximal values less than 10^{-3} were omitted, then LFB transformation was applied to the data. Scikit-learn's SVC was fitted to the first two resulting TCAM factors. Univariate time-series analysis was performed using lmer.

Dietary fiber intervention. 16S rDNA sequencing data and metadata for [11] were downloaded from ENA (project accession number: PRJNA560950). Overlapping paired-end FASTQ files of 16S amplicon sequencing data were matched and analyzed using the Qiime2 pipeline (q2cli version 2021.4.0) [12]. Poor quality bases were trimmed, sequences were denoised and binned to amplicon sequence variants (ASVs) using the dada2 plugin for Qiime2 [13]. Taxonomic assignment was performed using naïve Bayes feature classifier and Greengenes 13_8 database. Given the number of data points (n=34 participants), and under low rank assumption, CTF [3] was applied to the raw count data with target ranks ranging from one to ten, seeking to optimize the representation to show differences between groups. The analysis using TCAM was performed on top of two normalization methods: 1) a DFB transformed RCLR normalized data, where the RCLR was applied to the raw count data with target rank set to 7 (after searching for optimal rank, similarly to the procedure employed for CTF), 2) Relative abundance values were capped at 10^{-3}, i.e. features with maximal values less than 10^{-3} were omitted, then LFB transformation was applied to the data.
In all cases, PERMANOVA was applied to between-subjects distance matrices, computed in the embedding space using the minimal number of components such that the explained variance ratio is greater or equal to 0.2. Feature selection for univariate time-series analysis was done by taking the 0.75 quantile of loadings norm computed for the factors demonstrating significant univariate difference (ANOVA). Univariate time-series analysis was performed using lmer.

Identification of insulin resistance using longitudinal proteomics. Proteomics data and metadata were downloaded from https://figshare.com/articles/dataset/Multi_Omics_Seasonal_RData/12376508. Three years duration was stratified to trimesters, and repeated samples of the same participant within a trimester were median aggregated. Subjects lacking measurements in more than a single trimester were omitted from the analysis. Missing timepoints were imputed via linear interpolation or forward/backward filled in case of missing last/first trimester. The first year was considered as "baseline". Since proteomics data contain negative values, the data for each subject were shifted by the median baseline measurements for that subject.

Pediatric ulcerative colitis. Sample-specific metadata and final microbial OTU relative abundances were acquired from [14]. Subjects without at least one sample in each timepoint of the experiment (0, 4, 12, 52) were excluded. Relative abundance values were capped at 10^{-4}, i.e. features with maximal values less than 10^{-4} were omitted, and data from each participant were LFBtransformed. A multilayer perceptron (MLP) with single 1000 neuron wide hidden layer was trained to predict remission state using the minimal number of TCAM factors such that at least 80% of the variation in the data is explained by the factors. The same MLP architecture was trained using PCA for the log2-fold changes of weeks 12 and 52 from the baseline of each subject. Rocket based classifier was obtained by applying Rocket on top of the same pre-processing scheme, followed by logistic model (RidgeClassifier), where the hyper-parameters of both steps were optimized via a grid-search.

References

[1] Misha E. Kilmer, Lior Horesh, Haim Avron, and Elizabeth Newman. Tensor-tensor algebra for optimal representation and compression of multiway data. *Proceedings of the National Academy of Sciences*, 118(28):e2015851118, jul 2021.

[2] Alex H. Williams, Tony Hyun Kim, Forea Wang, Saurabh Vyas, Stephen I. Ryu, Krishna V. Shenoy, Mark Schnitzer, Tamara G. Kolda, and Surya Ganguli. Unsupervised Discovery of Demixed, Low-Dimensional Neural Dynamics across Multiple Timescales through Tensor Component Analysis. *Neuron*, 98(6):1099–1115.e8, jun 2018.

[3] Cameron Martino, Liat Shenhav, Clarisse A. Marotz, George Armstrong, Daniel McDonald, Yoshiki Vázquez-Baeza, James T. Morton, Lingjing Jiang, Maria Gloria Dominguez-Bello, Austin D. Swafford, Eran Halperin, and Rob Knight. Context-aware dimensionality reduction deconvolutes gut microbial community dynamics. *Nature Biotechnology*, pages 1–4, aug 2020.

[4] Omar Delannoy-Bruno, Chandani Desai, Arjun S. Raman, Robert Y. Chen, Matthew C. Hibberd, Jiye Cheng, Nathan Han, Juan J. Castillo, Garret Couture, Carlito B. Lebrilla, Ruteja A. Barve, Vincent Lombard, Bernard Henrissat, Semen A. Leyn, Dmitry A. Rodionov, Andrei L. Osterman, David K. Hayashi, Alexandra Meynier, Sophie Vinoy, Kyleigh Kirbach, Tara Wilmot, Andrew C. Heath, Samuel Klein, Michael J. Barratt, and Jeffrey I. Gordon. Evaluating microbiome-directed fibre snacks in gnotobiotic mice and humans. *Nature*, 595(7865):91–95, jul 2021.

[5] Frank L. Hitchcock. The Expression of a Tensor or a Polyadic as a Sum of Products. *Journal of Mathematics and Physics*, 6(1-4), 1927.

[6] Tamara G. Kolda and Brett W. Bader. Tensor decompositions and applications. *SIAM Review*, 51(3):455–500, August 2009.

[7] Ian T Jolliffe. Principal components in regression analysis. In *Principal component analysis*, pages 129–155. Springer, 1986.

[8] Ning Hao, Misha E. Kilmer, Karen Braman, and Randy C. Hoover. Facial recognition using tensor-tensor decompositions. *SIAM Journal on Imaging Sciences*, 6(1):437–463, January 2013.

[9] Nicholas A. Bokulich, Jennifer Chung, Thomas Battaglia, Nora Henderson, Melanie Jay, Huilin Li, Arnon D. Lieber, Fen Wu, Guillermo I. Perez-Perez, Yu Chen, William Schweizer, Xuhui Zheng, Monica Contreras, Maria Gloria Dominguez-Bello, and Martin J. Blaser. Antibiotics, birth mode, and diet shape microbiome maturation during early life. *Science Translational Medicine*, 8(343), June 2016.

[10] Cameron Martino*, Liat Shenhav*, Clarisse Marotz, George Armstrong, Daniel McDonald, Yoshiki Vázquez-Baeza, James T. Morton, Lingjing Jiang, Maria Gloria Dominguez-Bello, Austin D. Swafford, Eran Halperin, and Rob Knight. Context-aware dimensionality reduction deconvolutes gut microbial community dynamics. https://doi.org/10.24433/CO.5938114.v1, 2020.

[11] Edward C. Deehan, Chen Yang, Maria Elisa Perez-Muñoz, Nguyen K. Nguyen, Christopher C. Cheng, Lucila Triador, Zhengxiao Zhang, Jeffrey A. Bakal, and Jens Walter. Precision Microbiome Modulation with Discrete Dietary Fiber Structures Directs Short-Chain Fatty Acid Production. *Cell Host and Microbe*, 27(3):389–404.e6, mar 2020.

[12] Evan Bolyen, Jai Ram Rideout, Matthew R Dillon, Nicholas A Bokulich, Christian Abnet, Gabriel A Al-Ghalith, Harriet Alexander, Eric J Alm, Manimozhiyan Arumugam, Francesco Asnicar, Yang Bai, Jordan E Bisanz, Kyle Bittinger, Asker Brejnrod, Colin J Brislawn, C Titus Brown, Benjamin J Callahan, Andrés Mauricio Caraballo-Rodríguez, John Chase, Emily Cope, Ricardo Da Silva, Pieter C Dorrestein, Gavin M Douglas, Daniel M Durall, Claire Duvallet, Christian F Edwardson, Madeleine Ernst, Mehrbod Estaki, Jennifer Fouquier, Julia M Gauglitz, Deanna L Gibson, Antonio Gonzalez, Kestrel Gorlick, Jiarong Guo, Benjamin Hillmann, Susan Holmes, Hannes Holste, Curtis Huttenhower, Gavin Huttley, Stefan Janssen, Alan K Jarmusch, Lingjing Jiang, Benjamin Kaehler, Kyo Bin Kang, Christopher R Keefe, Paul Keim, Scott T Kelley, Dan Knights, Irina Koester, Tomasz Kosciolek, Jorden Kreps, Morgan GI Langille, Joslynn Lee, Ruth Ley, Yong-Xin Liu, Erikka Loftfield, Catherine Lozupone, Massoud Maher, Clarisse Marotz, Bryan Martin, Daniel McDonald, Lauren J McIver, Alexey V Melnik, Jessica L Metcalf, Sydney C Morgan, Jamie Morton, Ahmad Turan Naimey, Jose A Navas-Molina, Louis Felix Nothias, Stephanie B Orchanian, Talima Pearson, Samuel L Peoples, Daniel Petras, Mary Lai Preuss, Elmar Pruesse, Lasse Buur Rasmussen, Adam

Rivers, Michael S Robeson, II, Patrick Rosenthal, Nicola Segata, Michael Shaffer, Arron Shiffer, Rashmi Sinha, Se Jin Song, John R Spear, Austin D Swafford, Luke R Thompson, Pedro J Torres, Pauline Trinh, Anupriya Tripathi, Peter J Turnbaugh, Sabah Ul-Hasan, Justin JJ van der Hooft, Fernando Vargas, Yoshiki Vázquez-Baeza, Emily Vogtmann, Max von Hippel, William Walters, Yunhu Wan, Mingxun Wang, Jonathan Warren, Kyle C Weber, Chase HD Williamson, Amy D Willis, Zhenjiang Zech Xu, Jesse R Zaneveld, Yilong Zhang, Rob Knight, and J Gregory Caporaso. Qiime 2: Reproducible, interactive, scalable, and extensible microbiome data science. *PeerJ Preprints*, 6:e27295v1, oct 2018.

[13] Benjamin J Callahan, Paul J McMurdie, Michael J Rosen, Andrew W Han, Amy Jo A Johnson, and Susan P Holmes. DADA2: High-resolution sample inference from illumina amplicon data. *Nature Methods*, 13(7):581–583, May 2016.

[14] Melanie Schirmer, Lee Denson, Hera Vlamakis, Eric A. Franzosa, Sonia Thomas, Nathan M. Gotman, Paul Rufo, Susan S. Baker, Cary Sauer, James Markowitz, Marian Pfefferkorn, Maria Oliva-Hemker, Joel Rosh, Anthony Otley, Brendan Boyle, David Mack, Robert Baldassano, David Keljo, Neal LeLeiko, Melvin Heyman, Anne Griffiths, Ashish S. Patel, Joshua Noe, Subra Kugathasan, Thomas Walters, Curtis Huttenhower, Jeffrey Hyams, and Ramnik J. Xavier. Compositional and Temporal Changes in the Gut Microbiome of Pediatric Ulcerative Colitis Patients Are Linked to Disease Course. *Cell Host & Microbe*, 24(4):600–610.e4, oct 2018.
