# Supplementary figures and images for: Dimensionality reduction of longitudinal ’omics data using modern tensor factorizations
Source: PLoS Comput Biol. 2022 Jul 15;18(7):e1010212. doi: 10.1371/journal.pcbi.1010212 (PMC9328521; doi:10.1371/journal.pcbi.1010212)

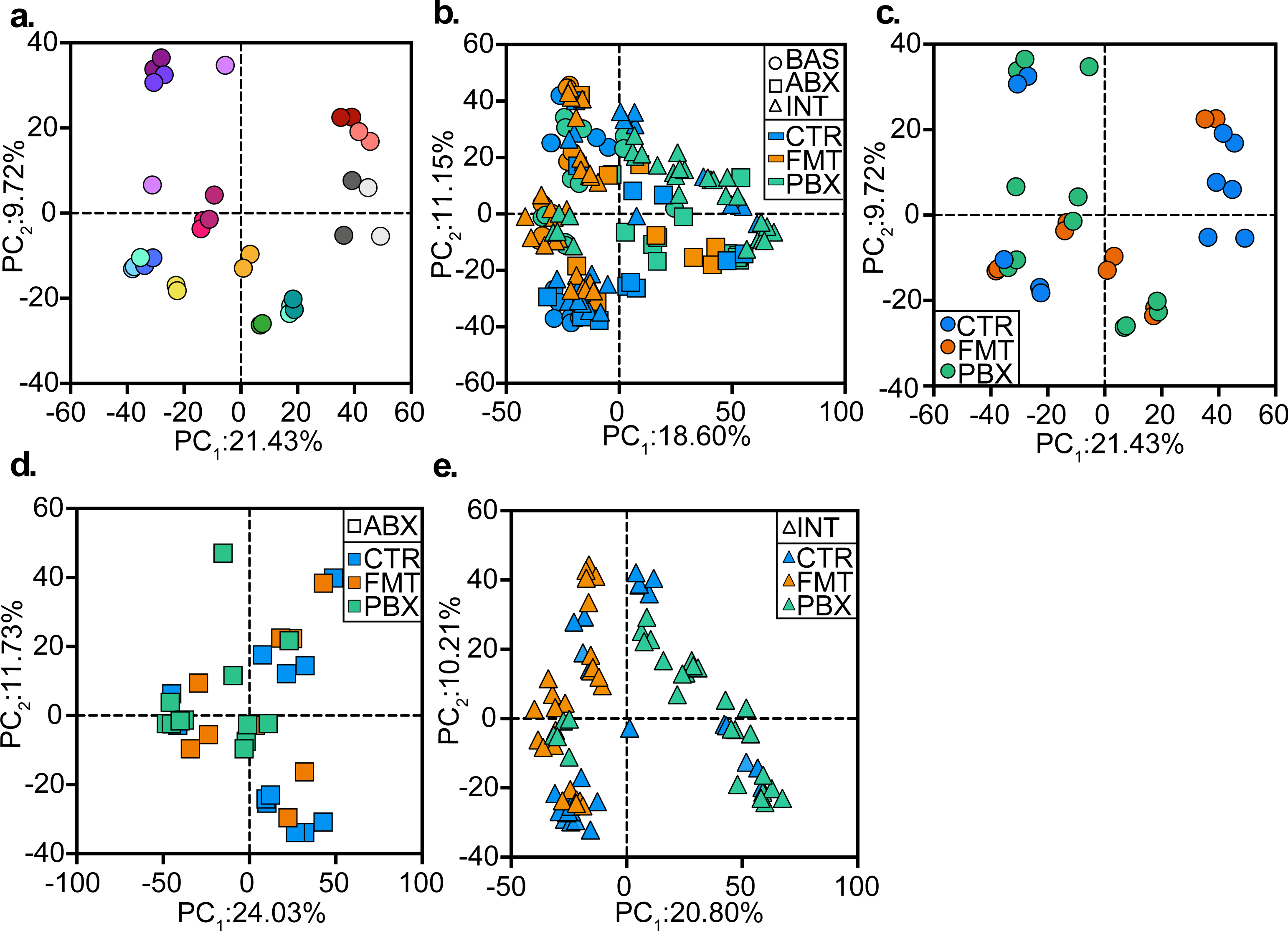

Supplement: S1 Fig — a PCA plot of baseline timepoints, 1–2 samples per each subject. Points are colored according to participant. b PCA plot of all timepoints. Points are colored according to group. c, d and e PCA plot of baseline, antibiotics, and intervention phases respectively. Points are colored according to group. (TIF) [file pcbi.1010212.s002.tif]

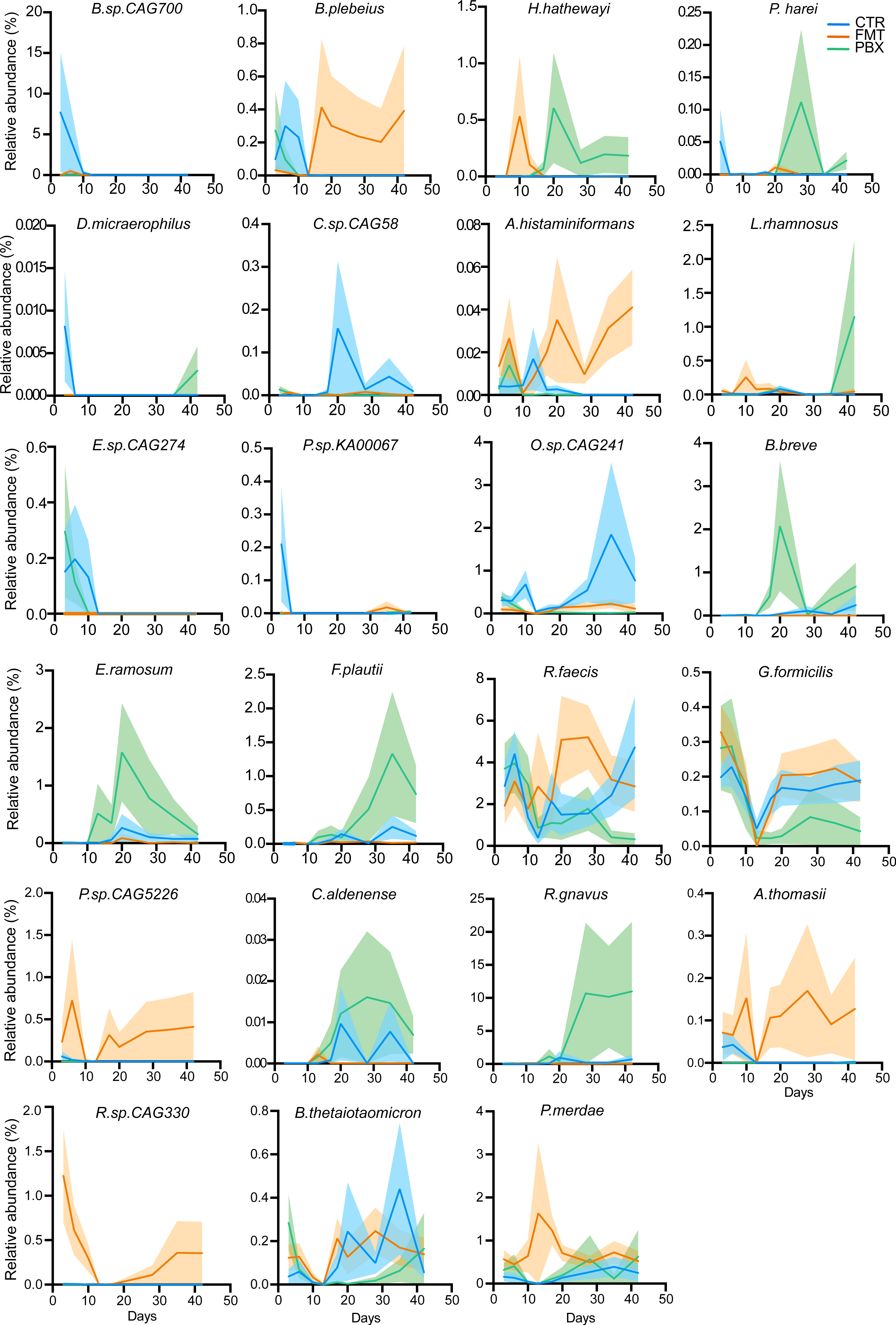

Supplement: S2 Fig — Time series of relative abundance levels for statistically significant taxa (q < 0.05, lmer), which were found using both tcam-based pruning and without any pruning strategy a, or when no pruning scheme was employed b. (TIF) [file pcbi.1010212.s003.tif]

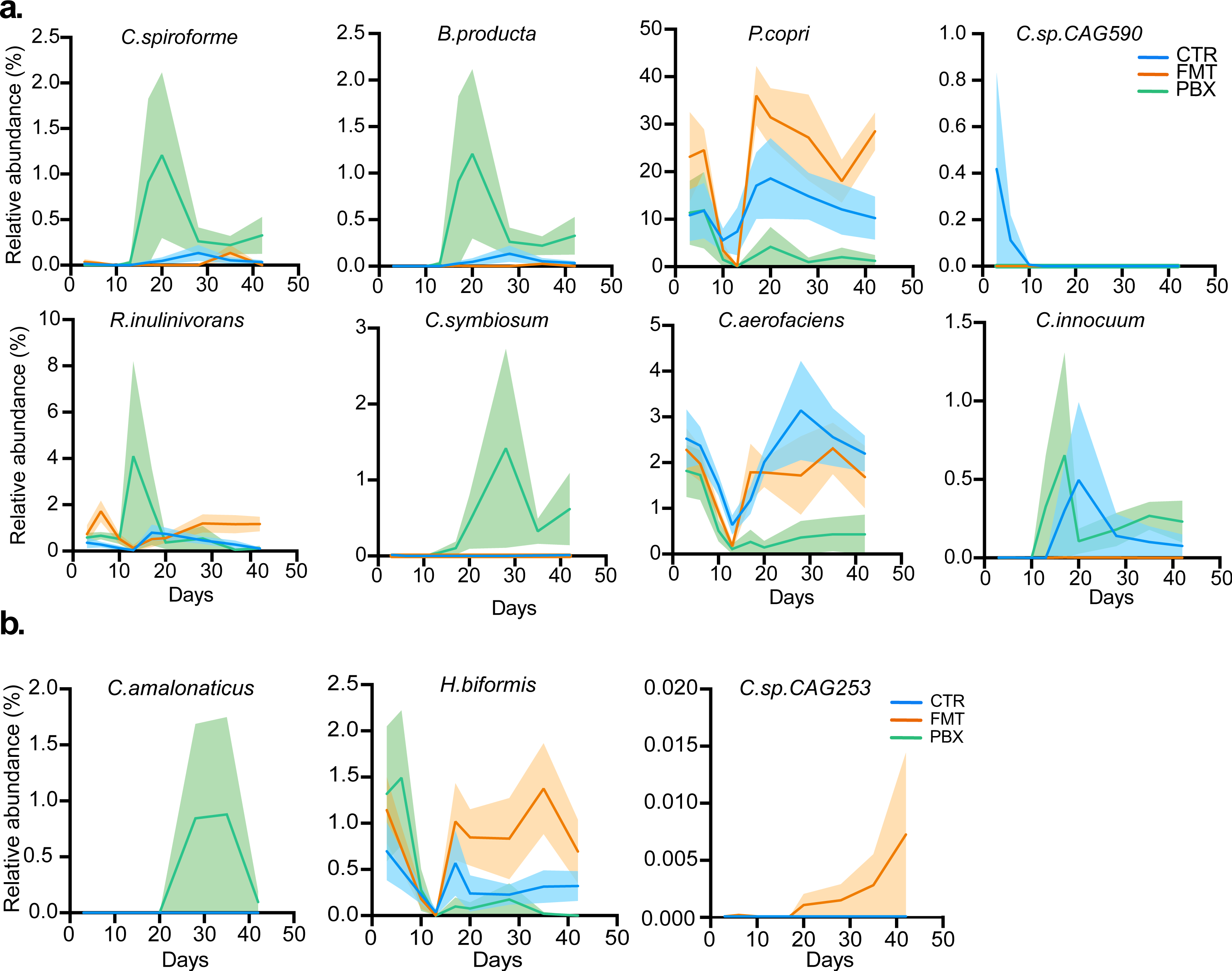

Supplement: S3 Fig — Time series of relative abundance levels for statistically significant taxa (q < 0.05, lmer), which were uniquely discovered when tcam based pruning of the features was used. (TIF) [file pcbi.1010212.s004.tif]

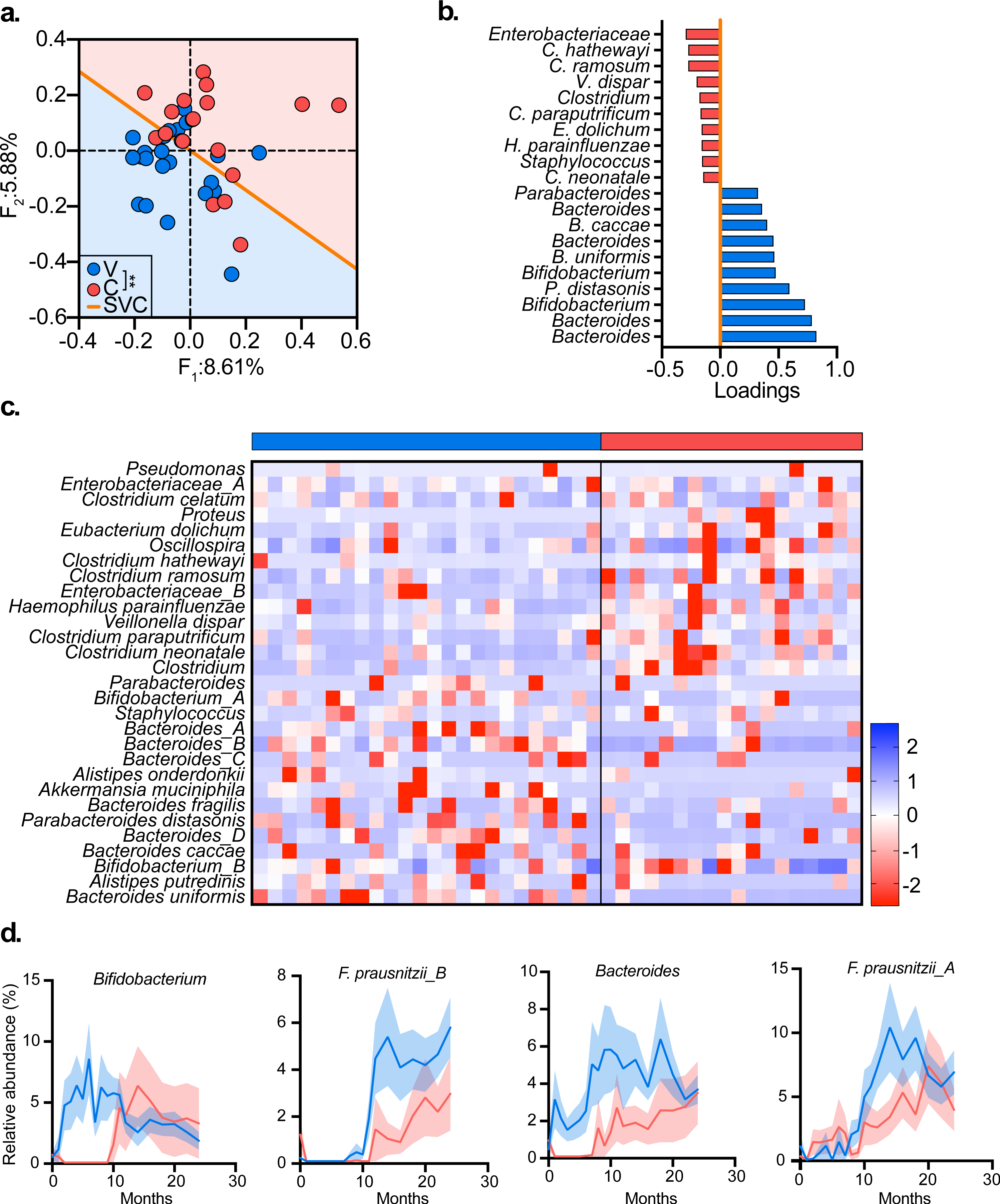

Supplement: S4 Fig — a Scatter plot of the first two tcam factors computed for the ECAM dataset [15]. Orange line and colored backgrounds show the boundary of decision and class domains computed using linear SVC; Inset; PERMANOVA. b Barplot showing the top 20 contributing features to the variation in orthogonal direction to the decision boundary. c Heatmap representing the cumulative change (iAUC) of the top 1% contributing features to the variation in orthogonal direction to the decision boundary; Color bar indicates z-score normalized value. d Time series describing relative abundances of bacteria with smallest adjusted p-value (lmer) and highest bacterial abundance after pruning strategy. (TIF) [file pcbi.1010212.s005.tif]

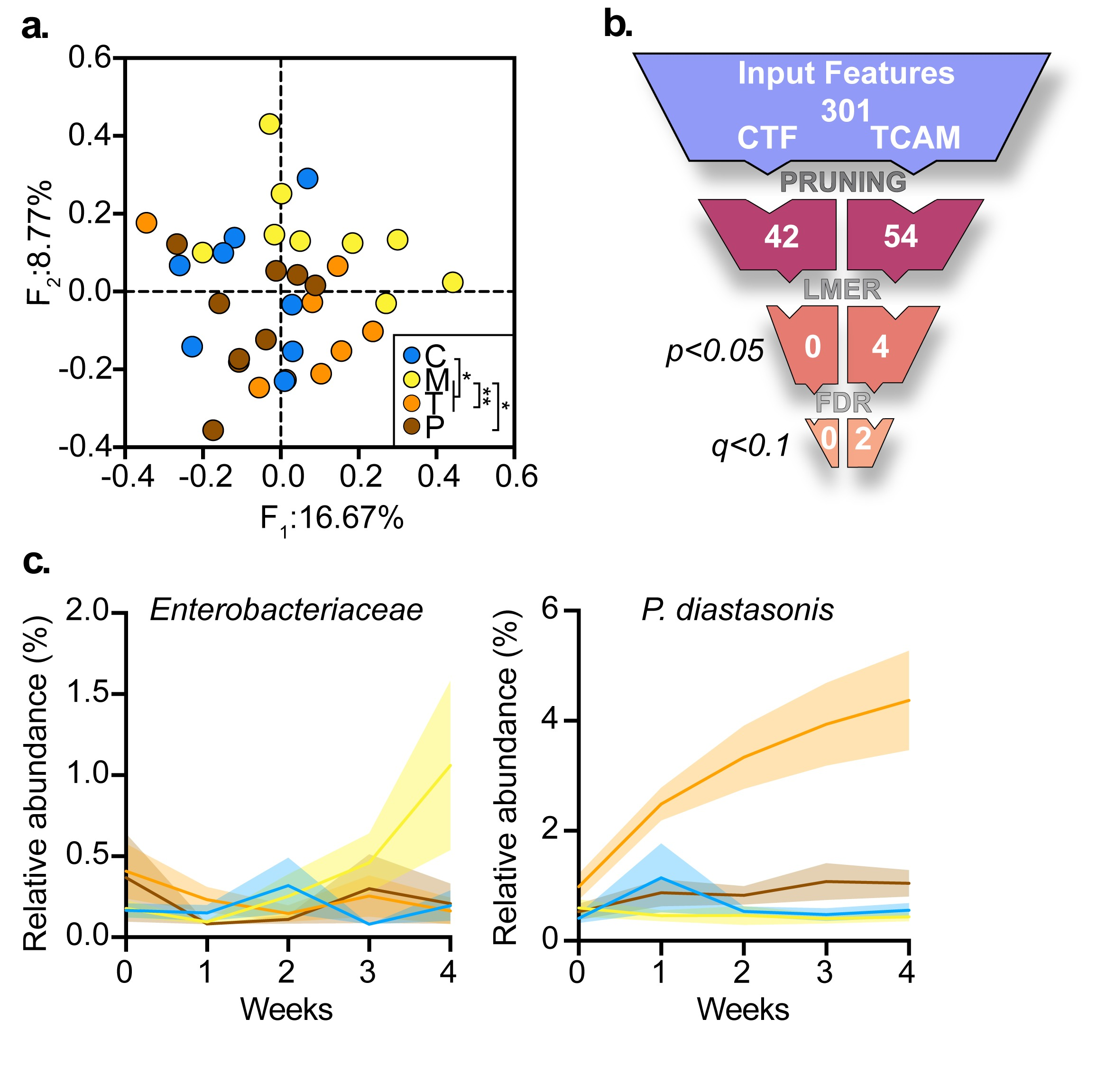

Supplement: S5 Fig — a Scatterplot showing the first factors acquired by employing tcam to the data of [16] following rclr normalization and DFB; Inset: Pairwise PERMANOVA. b Funnel showing the comparison between CTF (left) and tcam + rclr (right) as pruning strategies for univariate statistical hypothesis testing. c Time series describing all significant bacteria (lmer) found using rclr tcam based pruning strategy. (TIF) [file pcbi.1010212.s006.tif]

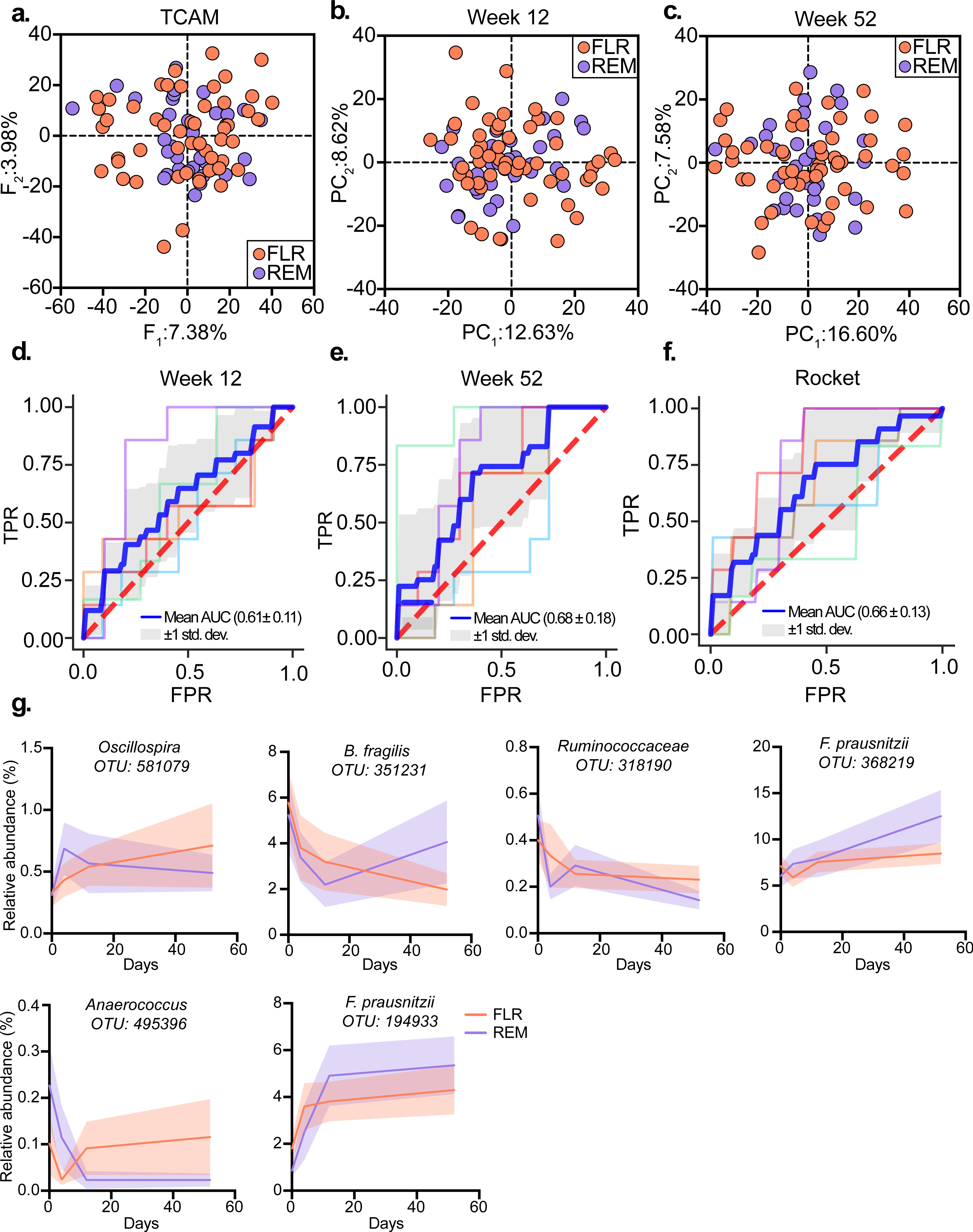

Supplement: S6 Fig — a, b, c Scatter plots for 2 leading factors of tcam for the whole dataset a PCA computed for log2 ratio of week 12 and baseline b PCA computed for log2 ratio of week 52 and baseline c Points are colored according to remission (REM) and flare (FLR) status. d and e ROC curve for MLP model trained to classify remission/flare based on PCA transformed log fold change between week 12 and baseline (d), log fold change between week 52 and baseline (e). f ROC curve for ridge-regression-classifier model trained to classify remission/flare given random kernel transformation (Rocket [24]) of the complete time-series. g Time series of relative abundance levels, highlighting the differences in trajectories of the features contributing to the remission status classification model. (TIF) [file pcbi.1010212.s007.tif]
