# Supplementary material for: Dimensionality reduction of longitudinal ’omics data using modern tensor factorizations
Source: PLoS Comput Biol. 2022 Jul 15;18(7):e1010212. doi: 10.1371/journal.pcbi.1010212 (PMC9328521; doi:10.1371/journal.pcbi.1010212)
Supplement: Attachment — Submitted filename: tcam_rev1_response.pdf [file pcbi.1010212.s008.pdf]

Dimensionality Reduction of Longitudinal 'Omics Data using Modern Tensor Factorizations.

April 19, 2022

Thank you very much for submitting your manuscript "Dimensionality Reduction of Longitudinal 'Omics Data using Modern Tensor Factorizations" for consideration at PLOS Computational Biology. As with all papers reviewed by the journal, your manuscript was reviewed by members of the editorial board and by several independent reviewers. The reviewers appreciated the attention to an important topic. Based on the reviews, we are likely to accept this manuscript for publication, providing that you modify the manuscript according to the review recommendations.
Both reviewers had some questions regarding the distinctive features and limitations of TCAM compared with some of the methods mentionned in your introduction. In particular, reviewer 1 had some concerns regarding the application of TCAM to feature engineering, which should be addressed in the revised version.
Please prepare and submit your revised manuscript within 30 days. If you anticipate any delay, please let us know the expected resubmission date by replying to this email.
When you are ready to resubmit, please upload the following:
[1] A letter containing a detailed list of your responses to all review comments, and a description of the changes you have made in the manuscript. Please note while forming your response, if your article is accepted, you may have the opportunity to make the peer review history publicly available. The record will include editor decision letters (with reviews) and your responses to reviewer comments. If eligible, we will contact you to opt in or out
[2] Two versions of the revised manuscript: one with either highlights or tracked changes denoting where the text has been changed; the other a clean version (uploaded as the manuscript file).
Important additional instructions are given below your reviewer comments.

Reviewer 1

This manuscript introduces TCAM, a tensor factorization method for the analysis of longitudinal omics data. The method is based on TSVDM, a generalized SVD for higher order tensors based on M-product framework. The authors showed that TCAM outperforms both matrix-based dimension reduction methods (i.e., PCA) and other tensor-based methods by mathematical formal analysis and real-world applications on metagenomics and proteomics data. Specifically, TCAM could capture the temporal intra-subject variations which matrix-based method could not do. Also, unlike other ALS-CP based tensor factorization methods, TCAM is proved to have optimal low rank approximation in terms of distortion and variance in the embedding space. TCAM can transform unseen data points to the embedding space and thus can be used as a feature engineering step in a ML workflow.

The manuscript is well structured and well written. While I was not able to confirm every single proof, I am confident that the topics are presented in a sound way and would make an interesting topic for the readers.

We thank the reviewer for supporting our paper.

1. The manuscript would however benefit from (1) presenting a concrete algorithm for TCAM similar to ALS algorithm for CP-based tensor factorization methods and (2) stating the computational complexity of the algorithm since higher order tensors could be suffered from the curse of dimensionality.

We thank the reviewer for these suggestions. We have incorporated in the models section Algorithms 1 and 2 which formally describe the process of constructing the TCAM for a given dataset and projecting points (multi-variate time-series) onto the learnt embedding subspace (respectively). In addition, we stated computational complexity analysis following the formal algorithms:
Algorithm 1 for obtaining TCAM highlights that most of the computational effort is due to the TSVDM, which requires $\mathcal{O}(n(pm^2+m^3))$ arithmetic operations, omitting the overhead involved with applying the $\times_3\mathbf{M}$ operations that are of negligent cost compared to that of the SVD computation.

2. For the application of TCAM to supervised ML, comparing TCAM with other single time-point methods is not strong enough to support TCAM as a feature engineering step. There are extensive techniques for feature ranking and selection for ML workflows. The author could compare TCAM with those methods to show how powerful TCAM is as a feature engineering method.

We thank the reviewer for this comment. In order to better showcase TCAM as feature engineering step, we now include an additional experiment in which we compare TCAM's performance with a classifier based on random convolutional kernel transform (Rocket, Dempster-2020) - a contemporary, highly competitive feature engineering technique whose performance when used as pre-processing step for time-series classification tasks were shown to be comparable with state-of-the-art time-series classification methods. The following text was added to the results section: ..., similarly to the performance of a time-series classification model based on random convolutional kernel transform (Rocket , Dempster-2020; AUROC=0.66)...

minor comment: One minor comment is: the citation should be in numeric order.

We now have reformatted the citations.

Reviewer 2

A novel tensor factorization method, TCAM, is proposed for analysis of longitudinal 'omics data. TCAM is derived from TSVDM, the tensor application of the popular SVD matrix factorization method. TCAM utilizes the right orthogonal tensor (V) and original data to create "scores" and "loadings" matrices describing the original data. New data can then be represented in the original data space using matrix/tensor products. In short, this method enables better evaluation of longitudinal data sets, and enables projection of new data into the existing space, enabling integration with machine learning algorithms. TCAM is demonstrated on an antibiotic intervention dataset and proteomics dataset, then described in a multilayer perceptron pipeline for a disease course dataset. The authors do an excellent job of describing the tensor factorization comprising their TCAM method (figure 1 is well done). I was also particularly impressed with figures 2-4 describing important equations. This work adequately defends their work as a valid approach to longitudinal 'omics analysis.

We thank the reviewer for supporting our paper.

The use cases are well-constructed and easy to follow, but

3. It would be interesting and potentially more convincing to see TCAM applied to denser or longer longitudinal datasets.

We thank the reviewer for this suggestion.Following his suggestion we added analysis of the longer and denser ECAM dataset, and indeed these additional analysis showcase TCAM's great power in simplifying and representing such data. The following text was added to the results section: To demonstrate the application of TCAM to the exploratory analysis of dense longitudinal time-series, we used the ECAM dataset Bokulich2016, which contains stool microbiome collected in high frequency during the first two years of life for 43 infants. In this example, we put our focus on the well characterized differences between development course of microbiomes of infants that were vaginally delivered (V), and those of infants delivered by cesarean section (C). Observing the leading TCAM factors of the data, we notice a significant differences between microbiome trajectories of the two modes, highlighted both by univariate test and a (linear kernel) support vector machine classifier (SVC) (SI 4a; PERMANOVA; $P < 0.01$). To find the bacteria of highest contribution to the separation between the temporal trajectories of the two modes of birth, we computed the magnitude of the feature loadings when projected onto the normal direction to the boundary of decision (SI 4b). Indeed, among the top 1% contributing features, we were able to identify bacteria such as *Bacteroides*, *Bifidobacterium* and *Enterobacteriaceae* that were previously found to exhibit differential behavior across time between the birth modes. Applying univariate tests following the same pruning strategy as in the post-antibiotics reconstitution example, resulted in a fine-grained, coherent view of the discriminatory features (SI 4c and 4d)

Supplemental data is robust and well done.

We thank the reviewer for supporting our work.

Questions/Content Revisions:

4. What (if any) are the limitations of TCAM? Would be good to address any limitations of applying TCAM in the discussion.

We apologize for what might have been perceived as 'overselling' of our work. To downtone our conclusions, we added the following texts to the revised discussion section:

- ... While distinct choices of **M** would generally result in different TCAM embeddings (and transformations), the explicit rank-q truncation of each resulting TCAM makes the q dimensional transform maximizing the variance and minimizing distortion in the algebraic framework defined by each **M**. One possible way to define the 'best' **M** for a given dataset, is the **M** for which the implicit rank of the (un-truncated) decomposition of the data is minimized. We consider this choice as the 'best' option as it surely provides the compressed most possible representation of the data. Alternatively, considering that any TSVDM (thus, any TCAM) may be written as approximation in CP form (Section 6.C, Kilmer2021), we get that the implicit rank under $\star_{\mathbf{M}}$ of the data equals, by definition, to its tensor-rank, making the task of finding the 'best' **M** equivalent to finding the tensor-rank of the data. Since tensor-rank computation constitutes a difficult problem in general Hillar2013, we conclude that our definition for the 'best' **M** is unhelpful as it is generally impossible to lay one's hands on. Yet, we have shown that when dealing with time-series data, taking **M** as the discrete cosine transformation, ...
  In that excerpt we discuss the requirement of choosing a domain transform, and resulting implications of different choices.
- While TCAM proves to constitute an useful tool for the analysis of longitudinal experimental designs, it relies on a fully sampled cohorts, i.e., where all participants provide a the same number of samples corresponding to similar time points. Even though missing data imputation is a classic use-case for low-rank approximations in general Martino2020,Williams2018 and there has recently been progress in the applications of TSVDM to incomplete data Qin2022, the accuracy and reliability of reconstructed data generally depends on assumptions regarding the generative process of the data, the frequency of observed values or their distribution across subjects, features and timepoints. Maintaining TCAM's universality to all kinds of 'omics data necessitates a detachment of the factorization from imputation and normalization of the data. Currently, prior to applying TCAM, it is up to the user to impute missing samples and normalize the data by any method that is appropriate to the data of interest. In this work, we have demonstrated TCAM's power in longitudinal 'omics data analysis while considering naive and straight-forward schemes for normalization and imputation (SI D).
  In that excerpt, we discuss the limitations of data normalization and missing data imputation that are detached from the factorization process and are under the user responsibility to take of prior to the application of TCAM

5. How would you suggest a user determine rank 'q' in truncated TCAM?

We thank the reviewer for this important question and now added the following explanation to the TCAM part in the models section: We now remark regarding the choice of q. The dimension of the reduced space depends on the purpose of the analysis. For example, one might choose q based on the traditional considerations for choosing number of principal components, such as signal - noise ratio assumptions or based on scree plots. Regardless of the considerations for choosing q, it should be noted that unlike CP, where the target rank of the approximation affects the resulting embedding, the TCAM factors are pre-determined by the data (and **M**), up to a variance and distortion invariant multiplication by a unitary tensor. Thus, the configuration reflected by the first i TCAM components remains unchanged when taking any $q \geq i$.

6. Is TCAM compatible with longitudinal datasets exhibiting missed samples? In other words, do all subjects need to have all samples?

This is an important question. One of TCAM's limitation is its inability of working with missing samples. This important limitation is now better disclosed in the discussion: While TCAM proves to constitute an useful tool for the analysis of longitudinal experimental designs, it relies on a fully sampled cohorts, i.e., where all participants provide a the same number of samples corresponding to similar time points. Even though missing data imputation is a classic use-case for low-rank approximations in general Martino2020,Williams2018 and there has recently been progress in the applications of TSVDM to incomplete data Qin2022, the accuracy and reliability of reconstructed data generally depends on assumptions regarding the generative process of the data, the frequency of observed values or their distribution across subjects, features and timepoints. Maintaining TCAM's universality to all kinds of 'omics data necessitates a detachment of the factorization from imputation and normalization of the data. Currently, prior to applying TCAM, it is up to the user to impute missing samples and normalize the data by any method that is appropriate to the data

of interest. In this work, we have demonstrated TCAM's power in longitudinal 'omics data analysis while considering naive and straight-forward schemes for normalization and imputation (SI D).

7. Many microbiologists use dissimilarity metrics (Bray-Curtis, Jaccard) for microbiome studies to measure sample relationships without placing too much faith in the sequencing output (known to be potentially biased). Besides centering via MDF, how else should data be prepared for TCAM to account for potentially flawed OTU counts?

The single constraint imposed on the data for guaranteeing TCAM's optimality properties is MDF. MDF follows normalization that is suitable for the data of interest and relevant to the scientific question underlying the experiment. Attempts to cover all possible normalization schemes for all types of existing 'omics methods while considering all possible aims of longitudinal study design would forever result in an incomplete recipe that, in our opinion, is likely to cause more harm than good by confusing the users with options irrelevant to their aims and goals. Thus, as a design choice, we decided to showcase TCAM's as the factorization utility that is completely detached from any pre-processing schemes. To stress out this point, we added the following to the discussion section:
...Maintaining TCAM's universality to all kinds of 'omics data necessitates a detachment of the factorization from imputation and normalization of the data. Currently, prior to applying TCAM, it is up to the user to impute missing samples and normalize the data by any method that is appropriate to the data of interest. In this work, we have demonstrated TCAM's power in longitudinal 'omics data analysis while considering naive and straight-forward schemes for normalization and imputation (SI D).

Is it possible to use distance/dissimilarity indices somewhere? I imagine not, since this would alter the format of the tensors (distances obviously become upper triangular matrices).

Albeit the close relations between PCA and PCoA (putting PCA as a special case of PCoA obtained by using Euclidean distance), higher order formulation of PCoA is not straightforward. For example, the 'dissimilarity tensor' may be defined as the 3rd order tensor $\mathfrak{D} \in \mathbb{R}^{m \times m \times n}$ whose entries $\mathfrak{D}_{i,j,t}$ denote the dissimilarity between subjects i and j at timepoint t, or alternatively, as the 4th order tensor $\mathfrak{D} \in \mathbb{R}^{m \times m \times n \times n}$ whose entry $\mathfrak{D}_{i,i',t,t'}$ holds the distance between subject i at timepoint t from subject i' at timepoint t'. Presenting tensor - PCoA would require the determination of proper definitions and theoretical work that justify these choices by implying statistical and algebraic guarantees implied by them, making this generalization an excellent research topic, but too broad for the scope of this work on TCAM. Nonetheless, we added to the the discussion section a discussion of future directions:
A probabilistic formulation for TCAM, i.e., as a model for generating high dimensional time-series data that is subjected to some prior assumptions imposed by the type of data, may also be useful in order to handle missing data and determination of uncertainty in estimates and predictions. Moreover, the tensor-tensor $\mathbf{M}$ product framework (Kilmer-2021), which is the theoretical foundation underlying TCAM, may be further utilized to produce additional factorization schemes, such as the decomposition of a dissimilarity tensors for microbiome applications, non-negative factorizations intended for count data and more.

8. What is the distinct advantage of TCAM compared to MetaLonDA, MiRKAT, SplinectomeR, etc. mentioned in the introduction? What is the distinct advantage of TCAM compared to MetaLonDA, MiRKAT, SplinectomeR, etc. mentioned in the introduction?

We apologise for this unclarity. MetaLonDA is a hypothesis - testing method. SplinectomeR, MiRKAT are regression tools. TCAM doesn't compete with such tools, but rather complements their use, as these methods are for the supervised pursuit of relationships between the data and observed traits, while TCAM extract components that are meaningful to the variation in an unsupervised matter (i.e. regardless of phenotype). We added the following content into the introduction in order to explain the differences between the aforementioned methods and tensor methods (TCA): Generally referred to as tensor component analysis (TCA) Williams2018, these multiway dimensionality reduction methods for omics data provide a view on global - multivariate variations in the data, thus complement methods such as Metwally2018, Shields-Cutler2018, Plantinga2017 for univariate time-series analysis.

In the Results, did you or original authors consider looking for taxa signals at other ranks (i.e. genera, family, class)?

Deehan et. al. considered all taxonomic ranks, yet the majority of dicussion in the original paper was focused on the species level. Suez et. al. thoroughly examined all taxa levels, and all mice

related results in this study are reported for level genus level and above. Schrimer et. al. focused only on the species level. In our experiments, we focus only on the species level.

9. Methods: Short paragraph describing MDF abruptly transitions to TSVDM then returns to mentioning MDF in TCAM part. Could rearrange or make this paragraph connect to TSVDM better. Results: (spelling correction) "Mutlivariate" in 2nd paragraph. Results: (spelling correction) "non of" in 4th paragraph

We appreciate the reviewer's attention and apologise for these glitches. The aforementioned paragraph was moved to the part discussing TCAM to form a more coherent text, and typos were corrected (including additional ones found when revising the text).
